# A Christianson syndrome-linked deletion mutation (∆^287^ES^288^) in SLC9A6 disrupts recycling endosomal function and elicits neurodegeneration and cell death

**DOI:** 10.1186/s13024-016-0129-9

**Published:** 2016-09-02

**Authors:** Alina Ilie, Andy Y. L. Gao, Jonathan Reid, Annie Boucher, Cassandra McEwan, Hervé Barrière, Gergely L. Lukacs, R. Anne McKinney, John Orlowski

**Affiliations:** 1 Department of Physiology, McGill University, Bellini Life Sciences Bldg., Rm, 166, 3649 Promenade Sir-William-Osler, Montreal, QC H3G 0B1 Canada; 2 Department of Pharmacology and Therapeutics, McGill University, Montreal, Canada

**Keywords:** NHE6/SLC9A6, Christianson syndrome, X-linked intellectual disability, Protein misfolding, Ubiquitination, Endosomal pH homeostasis, Membrane trafficking, Apoptosis

## Abstract

**Background:**

Christianson Syndrome, a recently identified X-linked neurodevelopmental disorder, is caused by mutations in the human gene SLC9A6 encoding the recycling endosomal alkali cation/proton exchanger NHE6. The patients have pronounced limitations in cognitive ability, motor skills and adaptive behaviour. However, the mechanistic basis for this disorder is poorly understood as few of the more than 20 mutations identified thus far have been studied in detail.

**Methods:**

Here, we examined the molecular and cellular consequences of a 6 base-pair deletion of amino acids Glu^287^ and Ser^288^ (∆ES) in the predicted seventh transmembrane helix of human NHE6 expressed in established cell lines (CHO/AP-1, HeLa and neuroblastoma SH-SY5Y) and primary cultures of mouse hippocampal neurons by measuring levels of protein expression, stability, membrane trafficking, endosomal function and cell viability.

**Results:**

In the cell lines, immunoblot analyses showed that the nascent mutant protein was properly synthesized and assembled as a homodimer, but its oligosaccharide maturation and half-life were markedly reduced compared to wild-type (WT) and correlated with enhanced ubiquitination leading to both proteasomal and lysosomal degradation. Despite this instability, a measurable fraction of the transporter was correctly sorted to the plasma membrane. However, the rates of clathrin-mediated endocytosis of the ∆ES mutant as well as uptake of companion vesicular cargo, such as the ligand-bound transferrin receptor, were significantly reduced and correlated with excessive endosomal acidification. Notably, ectopic expression of ∆ES but not WT induced apoptosis when examined in AP-1 cells. Similarly, in transfected primary cultures of mouse hippocampal neurons, membrane trafficking of the ∆ES mutant was impaired and elicited marked reductions in total dendritic length, area and arborization, and triggered apoptotic cell death.

**Conclusions:**

These results suggest that loss-of-function mutations in NHE6 disrupt recycling endosomal function and trafficking of cargo which ultimately leads to neuronal degeneration and cell death in Christianson Syndrome.

**Electronic supplementary material:**

The online version of this article (doi:10.1186/s13024-016-0129-9) contains supplementary material, which is available to authorized users.

## Background

Christianson syndrome (CS) is a recently described neurodevelopmental and progressively neurodegenerative disorder characterized by moderate to severe intellectual disability, epilepsy, mutism, truncal ataxia, hyperkinesis, happy demeanor, postnatal microcephaly and is often accompanied by one or more secondary symptoms (e.g., autistic behaviour, eye movement dysfunction, hypotonia, gastroesophageal reflux, low height and/or weight, high pain threshold, motor regression, cerebellar vermis and brain stem atrophy as well as neuronal cell loss) [1–3]; a phenotype that partially mimics Angelman syndrome (AS) [4, 5]. However, the underlying pathogenic gene variants are different. Whereas AS is caused by defects in the E3 ubiquitin ligase UBE3A/E6AP gene located at chromosome 15q11.2 [6, 7], CS was recently found to arise from mutations in the gene encoding the pH-regulating solute carrier (Na^+^, K^+^)/H^+^ exchanger NHE6/SLC9A6 located at chromosome Xq26.3 [2, 3, 8, 9]. The frequency of CS amongst X-linked developmental brain disorders has been estimated at approximately 1 to 2 % [3, 10–12], which places NHE6 amongst the more commonly mutated genes associated with X-linked intellectual disability (XLID) [13–17]. The descriptions of autistic behaviour in patients with CS also parallels recent findings of a significant reduction in NHE6 gene expression in postmortem cerebral cortex from patients with idiopathic autism compared to control tissue [18], further implicating an important role for this transporter in disorders of cognitive development. As with most X-linked disorders, mutations in NHE6 profoundly affect males whereas female carriers are either asymptomatic or display a milder phenotype [1, 3, 8].

NHE6 belongs to the endomembrane subclass of mammalian alkali cation/proton exchangers that are sorted differentially to discrete organelles along the secretory and degradative pathways [19, 20]. In the case of NHE6, it is widely expressed but most abundant in excitable tissues such as brain, heart, and skeletal muscle. In non-neuronal cells, the bulk of the transporter accumulates preferentially in a transferrin receptor (TfR)-enriched recycling endosomal compartment [21–23] where it has been implicated in the regulation of vesicular pH and trafficking [24, 25] and maintenance of epithelial cell polarity [24]. In the central nervous system, NHE6 is expressed in all regions and is particularly high in the hippocampus, cortex, and Purkinje cell layer of the cerebellum [26–28] (also see Allen Brain Atlas; http//mouse.brain-map.org; [29]). Within hippocampal neurons, NHE6 is present in both the soma and neurites, where it overlaps with known early and recycling endosomal markers and is especially prevalent in vesicles containing the glutamatergic α-amino-3-hydroxy-5-methyl-4-isoxazolepropionic acid receptor (AMPAR) subunit GluA1 [27] and the neurotrophin tropomyosin-related kinase receptor B (TrkB) which preferentially binds brain-derived neurotrophic factor (BDNF) and neurotrophin-4 (NT-4) [28]. Like AMPAR [30] and TrkB [31], NHE6 is recruited into dendritic spines (i.e., the postsynaptic compartment of the majority of excitatory synapses) upon induction of *N*-methyl-D-aspartate receptor (NMDAR)-dependent long term potentiation, thereby implicating its involvement in learning and memory processes [27]. In a NHE6 null mouse model, hippocampal neurons showed reduced axonal and dendritic arborization and decreased circuit activity [28] as well as a progressive loss of cerebellar Purkinje cells [26], further linking its participation in processes underlying neuronal growth, maturation, function and survival.

A variety of inherited and de novo mutations in NHE6 have recently been identified in CS patients, including frameshifts, nonsense, missense and deletions, although their precise consequences on neuronal function have yet to be elucidated [2, 3, 8, 9, 11, 12, 32–41]. The majority of these mutations cause premature translation termination and loss-of-function. However mutations that do not truncate the protein may have different consequences on its membrane targeting and activity which, in conjunction with other genetic factors, may account for the phenotypic diversity of patients with CS [3]. For example, Garbern et al. [9] described an in-frame excision of three amino acids (^370^Trp-Ser-Thr^372^, ΔWST) in the predicted ninth transmembrane helix of NHE6 that caused the protein to be largely retained in the endoplasmic reticulum (ER) [42]. While these patients displayed many of the pathophysiological and behavioural features ascribed to CS, they were distinguished by atypical accumulation of the microtubule protein tau in neuronal and glial cells of cortical and sub-cortical regions and widespread neuronal loss, features that are characteristic of adult-onset neurodegenerative disorders such as Alzheimer’s disease [9]. Patients with this mutation also exhibited only occasional mild microcephaly, modest gross motor disability and absence of the dysmorphic features attributed to the Christianson and Angelman-like syndromes.

Here, we examine one of the original CS mutations identified by Gilfillan et al. [8] that results in an in-frame deletion of two highly conserved amino acids (p.E255_S256del, ΔES) in the predicted seventh transmembrane helix of a short splice-variant of NHE6 (i.e., NHE6v2). This mutant variant was found to be unstable [43], though the precise mechanism of its pathogenicity remains poorly understood. In our study, we established relevant non-neuronal and neuronal cell model systems to gain greater insight into the molecular and cellular consequences provoked by this mutation, but using the equally abundant longest splice-variant of NHE6 (i.e., p.E287_S288del in NHE6v1) as a template for study. Our results show that though the mutant protein assembles properly as a homodimer and is sorted to the plasma membrane, its oligosaccharide maturation﻿, half-life and steady-state abundance are greatly reduced. In addition, the membrane trafficking of the ΔES mutant and certain other associated cargo are also impaired, ultimately eliciting cell death in both non-neuronal and neuronal cells.

## Methods

### Antibodies and reagents

Mouse monoclonal anti-hemagglutinin (HA) antibody was purchased from Covance Inc. (Berkeley, CA); rabbit polyclonal anti-HA, monoclonal anti-glyceraldehyde-3-phosphate dehydrogenase (GAPDH) and monoclonal anti-GFP antibodies were obtained from Abcam Inc. (Cambridge, MA); mouse monoclonal anti-Flag M2 antibody was from Sigma; rabbit polyclonal anti-GFP antibody was from Life Technologies. Mouse monoclonal anti-human transferrin antibody was from Invitrogen. Mouse monoclonal anti-β-tubulin was from Sigma. Mouse monoclonal anti-ubiquitin antibody (P4D1) was from Santa Cruz Biotechnology. Rabbit polyclonal anti-cleaved caspase-3 (Asp175) antibody was purchased from Cell Signaling Technology (kindly provided by Dr. Peter Siegel, McGill University). Horseradish peroxidase-conjugated secondary IgG antibodies, as well as FITC-conjugated goat anti-mouse secondary Fab were purchased from Jackson ImmunoResearch Laboratories (West Grove, PA). All Alexa Fluor® conjugated secondary antibodies were purchased from Molecular Probes (Eugene, OR). Alpha-minimum essential medium (α-MEM), fetal bovine serum, penicillin/streptomycin, and trypsin-EDTA were purchased from Wisent (Saint-Bruno, QC, Canada). The DMEM/F12 medium was from Corning. All other chemical and reagents were obtained from BioShop Canada (Burlington, ON, Canada), Sigma or Fisher Scientific and were of the highest grade available.

### Recombinant DNA constructs and mutagenesis

The long transcript splice-variant of human NHE6 (NHE6v1; NCBI refseq NM_001042537) was cloned from a human brain Matchmaker™ cDNA library (Clontech) using PCR methodology and was engineered to contain the influenza virus hemagglutinin (HA) (YPYDVPDYAS) epitope at its extreme C-terminal end. This construct was termed wild-type NHE6_HA_ (NHE6_HA_-WT) and inserted into the *HindIII* and *XbaI* sites of the mammalian expression vector pcDNA3 (Invitrogen), as described previously [42]. NHE6_HA_ was then used as a template to engineer the following mutations by PCR mutagenesis: double deletion mutation of amino acids E287 and S288 (ΔE287/S288, ΔES), the conservative double substitution E287Q/S288A, and the single mutations E287A, E287Q, and S288A.

The same template (NHE6_HA_) was also used to introduce a triple Flag epitope (AAA**DYKDDDDK**G**DYKDDDDK**G**DYKDDDDK**AAA) in the first extracellular loop immediately after residue Met^53^. First, PCR was used to engineer an in-frame *NotI* restriction site after M53, followed by the introduction of annealed primers representing the 3xFlag epitope, which generated a construct termed _3F_NHE6_HA_. This construct was further used as a template to introduce the ΔE287/S288, E287Q/S288A, E287Q, and S288A mutations using PCR mutagenesis.

Green fluorescent protein (GFP) C-terminal-tagged forms of NHE6 WT and ΔES mutant were constructed by insertion between the *XhoI* and *HindIII* restriction sites of the pAcGFP1-N1 vector (BD Biosciences Clontech, Palo Alto, CA). Insertion of the different epitope tags in the various positions did not alter the biochemical properties or cellular distribution of exogenous NHE6 compared to the endogenous protein [42]. All constructs were sequenced to insure that no additional mutations were introduced during PCR.

### Cell culture

Chinese hamster ovary AP-1 cells [44], HeLa, and HEK293 cells were cultured in α-MEM supplemented with 10 % fetal bovine serum, penicillin (100 units/mL), streptomycin (100 μg/mL), and 25 mM NaHCO_3_ (pH 7.4). Human neuroblastoma SH-SY5Y cells were cultured in high glucose Dulbecco’s Modified Eagle Medium (DMEM)/Ham’s F12 medium supplemented with 10 % fetal bovine serum.

Primary cultures of mouse hippocampal neurons were prepared from post-natal day (PD) 0–2 day C57BL/6 and L17 transgenic mice as previously described [27]. The L17 mice line express membrane-targeted enhanced GFP (mGFP) under the control of a Thy1.2 promoter cassette in a subset of hippocampal neurons, allowing the visualization of cell soma and other neuronal structures. To prepare cultures, the pups were decapitated, their brains were removed, and the hippocampi were dissected out. These hippocampi were maintained in chilled HBSS supplemented with 0.1 M HEPES buffer and 0.6 % glucose, then digested with 165 U papain for 20 min at 37 °C. Neurons and glia were dissociated by trituration and suspended in DMEM supplemented with 1 % penicillin-streptomycin, 10 % FBS, and 0.6 % glucose. Cells were then plated onto poly-D-lysine-coated 10 mm coverslips at an approximate density of 12,000 cells/cm^2^ and placed in an incubator at 37 °C. Twenty-four hours later, plating media was then replaced with Neurobasal-A growth media supplemented with 2 % B-27 supplement, 1 % GlutaMAX, and 1 % penicillin-streptomycin. Cultures were then fed every 3–4 days and maintained at 37 °C in a humidified environment of 95 % air, 5 % CO_2_.

### Western blotting

For western blot analyses, AP-1, HeLa or SH-SY5Y cells were grown in 10-cm dishes and transiently transfected with 5 μg of plasmid DNA encoding NHE6_HA_ wild-type or mutant constructs using Lipofectamine2000™ (Invitrogen) according to the manufacturer’s recommended procedure. Cell lysates were prepared following 6 to 48 h post-transfection (as indicated in the figure legends) by washing cells twice on ice with ice-cold PBS, followed by scraping in 0.5 mL of lysis buffer (0.5 % NP40/0.25 % sodium deoxycholate/PBS supplemented with protease inhibitor cocktail (Roche Diagnostics). Lysates were incubated for 30 min on a rocker at 4 °C, and then centrifuged for 20 min at 4 °C to pellet the nuclei and cellular debris. Twenty μg of protein from the resulting supernatants were eluted in sodium dodecyl sulfate (SDS)-sample buffer (50 mM Tris–HCl, pH 6.8, 1 % SDS, 50 mM dithiothreitol, 10 % glycerol, 1 % bromophenol blue), and subjected to 9 % SDS-polyacrylamide gel electrophoresis (SDS-PAGE), then transferred to polyvinylidene fluoride (PVDF) membranes (Millipore, Nepean, Ontario, Canada) for immunoblotting. The membranes were blocked with 5 % non-fat skim milk for 1 h, then incubated with the specified primary antibodies (mouse monoclonal HA 1:5000, ﻿anti-β-tubulin 1:10,000, anti-ubiquitin 1:2000, or GAPDH 1:50,000) in PBS containing 0.1 % Tween 20, followed by extensive washes and incubation with goat anti-mouse horseradish peroxidase (HRP)-conjugated secondary antibody for 1 h. Immunoreactive bands were detected using Western Lightning ^TM^ Plus-ECL blotting detection reagents (Perkin Elmer Inc., Waltham, MA).

### Endoglycosidase treatments

To obtain post-nuclear supernatants of AP-1 cells transiently expressing NHE6_HA_ WT or ΔES (24-h transfection), cells grown in 10-cm dishes were washed with ice-cold PBS, collected in 1 ml PBS, and then pelleted at 10,000 × *g* for 4 min at 4 °C. Pellets were resuspended in 300 μl sucrose solution (250 mM sucrose, 10 mM HEPES-NaOH, 1 mM EDTA, pH 7.5), and passaged 15 times through a 26.5-gauge needle. The nuclei and insoluble cell debris were sedimented at 700 × *g* for 15 min at 4 °C. The resulting post-nuclear supernatants were treated with endoglycosidases, according to the manufacturer’s recommendations. First, glycoproteins were denatured in 1x denaturing buffer (0.5 % SDS, 1 % β-mercaptoethanol) at 100 °C for 10 min. Samples were then divided equally and treated with either Peptide N-glycosidase F (PNGase F) (750 units, New England Biolabs, Mississauga, ON, Canada) or Endo-β-N-acetylglucosaminidase H (Endo H) (750 units, New England Biolabs, Mississauga, ON, Canada). The enzymes were added to 30 μl reactions and incubated overnight at 37 °C. Next day, samples were diluted with two-fold concentrated SDS-PAGE sample buffer, incubated for 30 min at room temperature, briefly centrifuged, and analyzed by SDS-PAGE and western blotting with monoclonal anti-HA antibody.

### Cell surface biotinylation

AP-1 cells expressing NHE6_HA_ WT or ΔES constructs were cultured in 10-cm dishes to sub-confluence, placed on ice and washed three times with ice-cold PBS containing 1 mM MgCl_2_ and 0.1 mM CaCl_2_, pH 8.0 (PBS-CM). Next, cells were incubated at 4 °C for 30 min with the membrane-impermeable reagent N-hydroxysulfosuccinimydyl-SS-biotin (0.5 mg/mL) (ThermoScientific, Rockford, IL). Cells were washed and incubated twice in quenching buffer (50 mM glycine in PBS-CM) for 7 min each on ice to remove unreacted biotin. After two more washes in PBS-CM, the cells were lysed for 30 min on ice, and then centrifuged at 16,000 × *g* for 20 min at 4 °C to remove insoluble cellular debris. A fraction of the resulting supernatant was removed and this represents the total fraction. The remaining supernatant was incubated with 100 μL of 50 % NeutrAvidin® Agarose Resin slurry (Fisher Scientific, Whitby, ON, Canada) in lysis buffer overnight at 4 °C to extract biotinylated membrane proteins. The proteins were then resolved by SDS-PAGE and analyzed by western blotting.

### Stability of NHE6 WT and ΔES mutant

To determine the stability of wild-type and mutant NHE6, AP-1 cells were grown in 10-cm dishes and transfected with NHE6_HA_ wild-type or ΔES mutant constructs. Twenty-four hours post-transfection, the cells were transferred to 6-well plates and further grown for 24 h. To inhibit new protein synthesis, cells were treated with cycloheximide (150 μg/mL) in α-MEM supplemented with 10 % FBS and penicillin/streptomycin for up to 24 h. At specific time points, cells were lysed, protein concentrations were measured, and equal quantities of protein were subjected to SDS-PAGE and immunoblotting with mouse monoclonal anti-HA and anti-GAPDH antibodies. The intensity of the bands was quantified by densitometry of X-ray films exposed in the linear range and analyzed using ImageJ software.

### Ubiquitination and protein degradation

To examine ubiquitination of NHE6 WT and ΔES, AP-1 cells expressing NHE6_HA_WT or ΔES were lysed 24 h after transfection in lysis buffer containing 10 mM N-ethylmaleimide (Sigma). Equal amounts of total protein were pre-cleared for 2 h on Protein G-Sepharose beads™ (GE Healthcare). After removing a small fraction of the pre-cleared cell lysate for immunoblotting, the remaining lysate was divided into two equal fractions: one fraction was used to immunoprecipitate the NHE6 protein overnight at 4 °C with a rabbit polyclonal anti-HA antibody (Abcam); the second fraction was subjected to immunoprecipitation with a nonspecific rabbit IgG antibody (Southern Biotech) as a negative control. Next day, the lysates were incubated with a 50 % slurry of Protein G-Sepharose beads for 3 h at 4 °C. The beads were subsequently washed and the immunoprecipitated proteins were eluted in sodium dodecyl sulfate (SDS)-sample buffer (50 mM Tris–HCl, pH 6.8, 1 % SDS, 50 mM dithiothreitol, 10 % glycerol, 1 % bromophenol blue). The immunoprecipitated proteins, along with aliquots representing the total lysate were subjected to 9 % SDS-polyacrylamide gel electrophoresis (SDS-PAGE), then transferred to polyvinylidene fluoride (PVDF) membranes (Millipore, Nepean, Ontario, Canada) for immunoblotting with a mouse monoclonal anti-ubiquitin antibody (Santa Cruz Biotechnology). The membranes were then stripped and reblotted with a mouse monoclonal anti-HA antibody (Covance).

AP-1 cells were cultured in 3.5-cm dishes and transfected with NHE6_HA_WT or ΔES using Lipofectamine2000™ (Invitrogen). Twenty-four hours after transfection, cells were treated with DMSO as control, the proteasomal inhibitors MG-132 (40 μM) or lactacystin (30 μM), the lysosomal inhibitors leupeptin/pepstatin (100 μg/ml) or chloroquine (500 μM), all in the presence of cycloheximide (150 μg/mL) to prevent new protein synthesis. Cellular lysates were obtained after 4 and 8 h of treatment and equal amounts of proteins were subjected to SDS-PAGE and western blotting with a mouse monoclonal anti-HA antibody. The membranes were also immunoblotted with mouse monoclonal anti-GAPDH antibody as a loading control.

### Co-immunoprecipitation

To test the hypothesis that WT and ΔES mutant can heterodimerize, HeLa cells were cultured in 10-cm dishes and transfected with a total of 8 μg of expression plasmid DNA containing NHE6_HA_WT, NHE6v1_GFP_ΔES or empty vector (pCMV), either singly or in combination (4 μg each), using FuGene6 (Promega), according to the manufacturer’s instructions. Forty hours post-transfection, lysates were prepared by washing the cells twice with ice-cold PBS and scraping them into 500 μl of cell lysis buffer (0.5 % Nonidet-P40, 0.25 % sodium deoxycholate, and protease inhibitors in PBS, pH 7.4). Cell lysates were rocked at 4 °C for 30 min and centrifuged for 20 min at 16,000 × *g* to pellet cellular debris. Supernatants were pre-cleared for 2 h on Protein G-Sepharose beads™ (GE Healthcare). The beads were removed by brief centrifugation and a fraction of the cell lysate was removed for immunoblotting. Rabbit polyclonal anti-HA antibody (5 μg) was added to the remaining cell lysates and incubated with gentle rocking overnight at 4 °C. Subsequently, a 50 % slurry of Protein G-Sepharose beads was added to each tube and incubated with the immunoprecipitates for 2 h at 4 °C. The immunoprecipitated proteins, as well as aliquots of initial lysates were resolved by SDS-PAGE, transferred to polyvinylidene fluoride membranes, and immunoblotted with the indicated mouse monoclonal antibodies.

### Fluorescence-based endocytosis assay

AP-1 cells were grown in 10-cm dishes and transiently transfected with 6 μg of _3F_NHE6_HA_ WT or ΔES DNA constructs. Twenty-four h post-transfection, cells were transferred to 12 well-plates and grown for an additional 12 to 24 h. Cells were chilled on ice, washed with ice-cold PBS-CM, pH 7.4, blocked in 10 % goat serum/PBS-CM, and then incubated with a mouse monoclonal anti-Flag antibody (1:3000) (Sigma) on ice. Internalization of the bound antibody was initiated by incubating the cells with warm (37 °C) α-MEM for the indicated time points and terminated by placing the plates on ice. Cell were washed and labeled with goat anti-mouse HRP-conjugated secondary antibody (1:1000) (GE Healthcare). After extensive washes with PBS-CM, cells were treated on ice with Amplex® Red reagent (Invitrogen). Aliquots were transferred to 96-well plates and fluorescence readings were taken with a POLARstar OPTIMA (BMG Labtech. Inc, Offenburg, Germany) plate reader using 544-nm excitation and 585-nm emission wavelengths. All experiments were performed in triplicates and repeated at least three times. Results were expressed as a percentage of the fluorescence recorded prior to internalization, after subtraction of the value measured with mock-transfected cells. Results are shown as mean ± standard error of the mean (S.E.M.).

### Measurement of endosomal pH

AP-1 cells were transfected with _3F_NHE6_HA_WT or ΔES and cell surface resident proteins were labeled on ice with mouse monoclonal anti-Flag antibody (1:2000 dilution, Sigma-Aldrich) and FITC-conjugated goat anti-mouse secondary Fab fragment (1:1000 dilution, Jackson ImmunoResearch Laboratories, West Grove, PA). Cells were then washed in PBS-CM and chased for the indicated time points in growth medium at 37 °C. Endosomal pH was measured by single-cell fluorescence ratiometric imaging analysis (FRIA) on an Axiovert 100 inverted fluorescence microscope (Carl Zeiss MicroImaging, Toronto, ON, Canada) at room temperature equipped with a Hamamatsu ORCA-ER 1394 (Hamamatsu, Japan) cooled CCD camera and a Planachromat (63 × NA 1.4) objective essentially as described previously [45]. Image acquisition and FRIA were performed with MetaFluor software (Molecular Devices, Downingtown, PA). Images were acquired at 490 ± 5 and 440 ± 10 nm excitation wavelengths, using a 535 ± 25-nm emission filter. In each experiment, the pH of 100–1000 vesicles was determined. Mono- or multipeak Gaussian distributions of vesicular pH values were obtained with Origin 7.5 software (OriginLab, Northampton, MA). Calibration curves have been performed as previously described [45]. Briefly, *in situ* calibration was performed by clamping the vesicular pH between 4 and 7.5 in K^+^-rich medium (135 mM KCl, 10 mM NaCl, 20 mM Hepes or 20 mM MES, 1 mM MgCl_2_, and 0.1 mM CaCl_2_) with 10 μM nigericin, 10 μM monensin, 0.4 μM bafilomycin and 20 μM carbonyl cyanide m-chlorophenyl hydrazone (CCCP) and recording the fluorescence ratios.

### Calcium-phosphate transfection

Primary neurons were transfected by calcium phosphate-mediated transfection [46]. Briefly, at 10–12 DIV, coverslips were transferred into a 35-mm dish filled with warmed preconditioned growth media. For four coverslips, 4 μg of DNA plasmid was mixed with 50 μl 250 mM CaCl_2_ solution, which was then added to 50 μl 2x HEPES-buffered phosphate solution, pH 7.05 to form DNA-tagged calcium phosphate precipitate. This was added dropwise to each dish of coverslips, which were then incubated at 37 °C for 90 min. Afterwards, 33 μl of 0.3 M MES hydrate solution, pH 5.5, was added to each dish, which acidified the media to dissolve any remaining precipitate. The coverslips were returned to their original plates with fresh growth media, and cultures were then maintained at 37 °C for 48 h before being processed and fixed.

Cultures prepared from C57BL/6 mice were transfected with GFP and NHE6_ChFP_WT and NHE6_ChFP_∆ES. The L17 cultures, which already express myristoylated GFP (mGFP), were instead transfected with NHE6_ChFP_WT and NHE6_ChFP_∆ES and incubated with Tf-AF^633^ (100 μg/ml) for 1 h at 37 °C and then fixed.

### Immunofluorescence confocal microscopy

To examine the internalization of NHE6 into transferrin-containing endosomes, AP-1 cells were grown in 10-cm dishes and transfected with _3F_NHE6_HA_ WT or ΔES mutant. Twenty-four hours post-transfection, the cells were transferred to fibronectin-coated glass coverslips and grown for an additional 12 to 24 h (as specified in text). For the zero time point, cells were serum-depleted for 1 h, incubated with 10 μg/ml Alexa Fluor® 488-conjugated transferrin (Tf-AF^488^) for 45 min in serum-free media at 37 °C, and then placed on ice. Cells were then labelled for 1 h on ice with mouse monoclonal anti-Flag antibody (1:2000) in PBS-CM/10 mM glucose/10 mM HEPES, pH 7.4, washed 3 times with PBS supplemented with 1 mM MgCl_2_ and 0.1 mM CaCl_2_ (PBS-CM), and then incubated on ice with goat anti-mouse Alexa Fluor® 568-conjugated secondary antibody (1:1300). After extensive washes with PBS, cells were fixed in 2 % paraformaldehyde/PBS for 20 min at room temperature and mounted onto glass slides. For the 60 min endocytosis time point, after serum depletion, cells were labeled on ice with the primary anti-Flag and secondary Alexa Fluor® 568-conjugated antibodies, and then chased with serum free media at 37 °C for 1 h. For transferrin labelling, the chasing media contained 10 μg/ml Tf-AF^488^ during the last 45 min of chase. Cells were subsequently fixed and mounted. Cells were examined by laser scanning confocal microscopy using the ZEN software of a Zeiss LSM 710 Meta equipped with a PMT detector, with images acquired using a 63 × /1.4 N.A. oil immersion objective lens.

Primary hippocampal neuronal cultures were fixed with 4 % PFA/0.1 M PB, pH 7.4 (Sigma Aldrich) for 15 min at room temperature and washed with 0.1 M PB. C57BL/6 cultures immunoprocessed for cleaved caspase-3 (cCASP3) were first permeabilized for 1 min in 0.2 % Triton X-100/0.1 M PB and blocked for 1 h at room temperature in 0.2 % Triton X-100/1 % HIHS/0.1 M PB before being incubated with a primary rabbit polyclonal antibody against cleaved caspase-3 (Asp175) (cCASP3) (Cell Signaling Technology, 1:300) diluted in blocking solution overnight at 4 °C. After subsequent washing, cells were incubated with a secondary goat anti-rabbit DyLight 649-conjugated secondary antibody (Jackson laboratories, 1:1000) diluted in 1 % HIHS/0.1 M PB for 45 min at room temperature, washed again, and mounted. All coverslips were mounted onto SuperFrost (Menzel-Glaser) microscope slides using UltraMount fluorescence mounting medium (Dako) and left to dry overnight at room temperature in the dark. The soma of transfected neurons were then examined for cCASP3 staining to assess apoptosis.

Mounted primary hippocampal cultures were imaged using a Leica SP2 confocal microscope. Images were acquired using 40X and 63X HCXPL APO oil-immersion objectives (NAs 1.25 and 1.4, respectively). GFP was imaged using a 488 nm Ar laser line; mCherry was imaged using a 543 nm HeNe laser line, and Alexa Fluor 647, Alexa Fluor 633 and DyLight 649 were imaged using the 633 nm HeNe laser line. Channels were acquired sequentially to prevent spectral overlap of fluorophores. Optical sections of 300–500 nm were taken and frame averaged 3X at low resolution or line-averaged 2X at high resolution to improve the signal-to-noise ratio. Images were first deconvolved using Huygen’s Essential software by using a full maximum likelihood extrapolation algorithm (Scientific Volume Imaging), and 3D images were compiled as maximum intensity projections using Imaris software (Bitplane Ag). Colocalization analyses between NHE6 and AF-Tfn were determined using the ImarisColoc algorithm, which generated a new channel (coloc) containing voxels representing pixels of channel overlap. This also automatically calculated Mander’s coefficient M1-M2 values between NHE6 and Tf-AF^633^ channels from a set threshold. Thresholds were applied relatively consistently between cells in order to remove subjectivity during the analysis.

Topographical order of neuronal morphology was performed on 3D confocal images of primary hippocampal neurons prepared from C57BL/6 mice. Images were analyzed using the FilamentTracer program (Bitplane AG, Zurich, Switzerland), which semi-automatically detects 3D neuronal GFP-labeled filament structures and calculates parameters such as the number of branch points, total dendrite length and area.

### Flow cytometry

To measure transferrin and EGF uptake by flow cytometry, HeLa cells were transfected with GFP alone, NHE6_GFP_ WT or ΔES mutant using FuGene6 (Promega). Forty-eight h after transfection, the cells were serum-depleted for 2 h, and then incubated with Alexa Fluor® 633-conjugated transferrin (Tf-Alexa^633^, 10 μg/mL) or Alexa Fluor® 647-conjugated EGF (EGF-Alexa^647^, 100 ng/ml) for 5 min at 37 °C, in the absence or presence of 10-fold excess unlabelled transferrin or EGF, respectively, followed by washes to remove unbound transferrin or EGF. Cells were detached from the plates by trypsinization and 5 μL of the cell viability dye 7-amino-actinomycin D (7-AAD, eBioscience) was added to each cell suspension. Cells were analyzed by flow cytometry using a FACS Aria Sorter (Becton Dickinson, San Jose, CA). A gate was set around the GFP-positive cells and the amount of Tf-Alexa^633^ or EGF-Alexa^647^ taken up by 10^4^ GFP-expressing live cells (i.e., 7-AAD negative) was measured using the BD FACS Diva software.

### siRNA Knockdown

HeLa cells were cultured in 6-well plates and transfected with 100 nM control siRNA pool #1 (scrambled siRNA) or SMARTpool® NHE6 siRNA (Dharmacon, Lafayette, CO) using Dharmafect1 transfection reagent (Dharmacon) according to the manufacturer’s recommended protocol. Seventy-two hours post-transfection, the cells were serum-starved for 1.5 h and then incubated with Tf-AF^633^ for 20 min at 37 °C. 10^5^ cells were analyzed by flow cytometry per experiment.

### Apoptosis assays

Apoptosis was measured using three independent methods: (1) a flow cytometry assay that measures changes in plasma membrane asymmetry (annexin V-allophycocyanin conjugate (Annexin V-APC) binding to phosphatidylserine) and permeability (propidium iodide, PI); (2) a luminescent assay to detect caspase 3/7 activity; and (3) an immunofluorescence-immunocytochemistry assay to detect activated cleaved caspase-3 (Asp175).

For flow cytometry, AP-1 cells were grown in 6-cm dishes and transfected with 4 μg of GFP, NHE6v1_GFP_ WT or ΔES using Lipofectamine2000 (Invitrogen). Forty-eight h post-transfection, the cells were washed twice with warm (37 °C) PBS, detached using Cell Dissociation Buffer (GIBCO), and then collected by centrifugation. The cell pellets were re-suspended in PBS and labeled using the Annexin V-APC Apoptosis Detection Kit (eBioscience) according to the manufacturer’s instructions. After labeling, cells were placed on ice, and 1 × 10^4^ GFP-positive cells were examined on a BD™LSR II flow cytometer and the percentage of AnnexinV-positive cells (i.e., early apoptotic), PI-positive (i.e., necrotic), and AnnexinV/PI – double positive cells (i.e., late apoptotic), was determined.

Caspase 3/7 activity was measured using the luminescent Caspase-Glo® 3/7 assay (Promega). AP-1 cells were transfected in 10 cm dishes with 8 μg of GFP vector, NHE6v1_GFP_ wild-type or ΔES using Lipofectamine2000 (Invitrogen). Twenty-four hours after transfection, cells were sorted using a Becton Dickinson FACSAria Sorter and 5 × 10^4^ GFP-positive cells were plated in each well of a 96-well plate. Twenty-four hours later, the assay was performed according to the manufacturer’s instructions and luminescence was read in white-walled 96-well luminometer plates.

To measure apoptosis in primary hippocampal neurons, immunocytochemistry was performed on fixed neurons transfected with cytosolic GFP alone or co-transfected with GFP and NHE6v1_ChFP_ WT or ΔES using a primary rabbit polyclonal antibody against cleaved caspase-3 (Asp175) (cCASP3) (Cell Signaling Technology, 1:300) and a secondary goat anti-rabbit DyLight 649-conjugated secondary antibody (1:1000) from Jackson laboratories. The soma of transfected neurons were then examined for cCASP3 staining to assess apoptosis.

### Real-time PCR (RT-PCR)

HeLa cells were grown in 6-well plates and transfected with 100 nM siRNA control pool #1 (scrambled siRNA) or SMARTpool® NHE6 siRNA (Dharmacon) using Dharmafect1 transfection reagent (Dharmacon) according to the manufacturer’s recommended protocol. Total RNA was extracted with TRIzolreagent (InvitrogenCanada Inc.). Real-time PCRs were performed using IQ SYBR Green Supermix (Bio-Rad Laboratories Inc., ON) and analyzed with Gene Expression Analysis for iCycler iQ® Real-Time PCR Detection System (BioRad).

### Statistical analyses

The data represent the mean ± the standard error of the mean (S.E.M.) and statistical analyses were performed by using the Student’s *t*-test or a one-way analysis of variance (ANOVA) followed by a Tukey test. A minimum *p*-value of <0.05 was considered significant.

## Results

### Post-translational processing and stability of NHE6ΔES is impaired

**Fig. 1:**
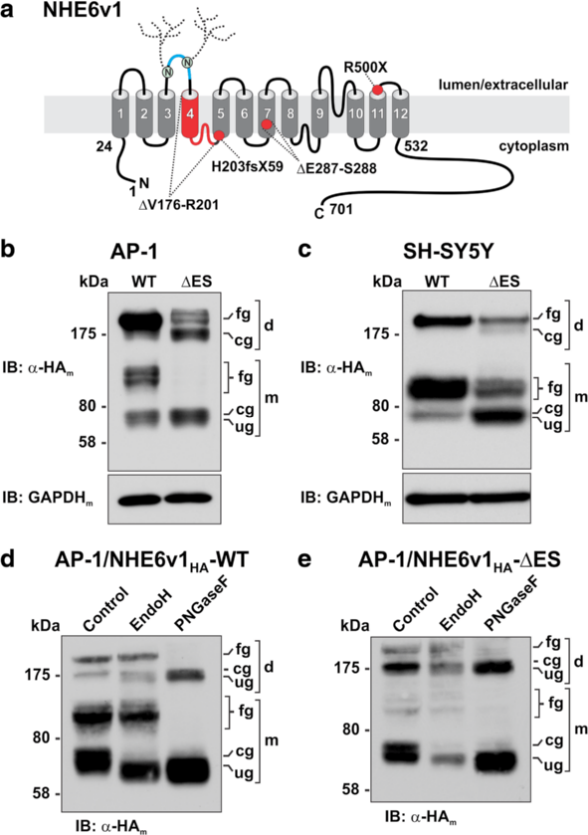
Expression and post-translational processing of NHE6 ΔES mutant protein is impaired in transfected AP-1 and SH-SY5Y cells. **a** Schematic drawing of the predicted membrane topology of mammalian NHE6v1 (based on sequence alignment and transmembrane organization of NHE1 proposed by Wakabayashi et al. [127] and Nygaard et al. [128]) and locations of mutations (*red shading*) identified by Gilfillan et al. [8] in patients with Christianson syndrome. The *blue shading* in the second extracellular loop (EL2) represents the additional 32 amino acids (residues 145–176) present in the NHE6v1 splice-variant. Two predicted *N*-glycosylation sites within EL2 are also illustrated. **b** AP-1 and **c**, SH-SY5Y cells were transiently transfected (24 h) with NHE6_HA_ WT or ΔES mutant. Total cell lysates of WT and ΔES-transfectants were examined by SDS-PAGE. The immunoblots were probed with a mouse monoclonal anti-HA antibody (α-HA_m_) to detect NHE6v1. NHE6v1 migrates as multiple bands: slower migrating high molecular weight bands representing the fully-glycosylated (*fg*) and core-glycosylated (*cg*) dimeric forms of the exchanger (~200 and 175 kDa, respectively) that do not fully dissociate under SDS-PAGE conditions and faster migrating fully-glycosylated (*fg*, ~100 kDa) and core-glycosylated (*cg*, ~70 kDa) and unglycosylated (*ug*, ~65 kDa) forms of the monomeric protein. To control for protein loading, the blots were reprobed with a mouse monoclonal anti-GAPDH antibody (α-GAPDH_m_). **d**, **e** To confirm the nature of the oligosaccharide modifications of the NHE6 bands, AP-1 cells transiently transfected (24 h) with WT or ΔES constructs were lysed in non-detergent buffers and post-nuclear supernatants were left untreated or incubated with either endoglycosidase H (EndoH), which cleaves only asparagine-linked mannose-rich oligosaccharides (i.e., core-glycosylated) but not more highly processed complex oligosaccharides (i.e., fully-glycosylated), or peptide-N-glycosidase F (PNGaseF) which cleaves between the innermost N-acetylglucosamine and asparagine residues of all oligosaccharide structures (i.e., high mannose, hybrid, and complex). The lysates were then subjected to SDS-PAGE and immunoblotting with an α-HA_m_ antibody. The data are representative of three independent experiments

A foundational study by Gilfillan et al. [8] identified four mutations in NHE6 (ΔV176-R201, H203fsX59, ΔE287-S288, R500X in the longest splice-variant of NHE6, NHE6v1, as illustrated in Fig. 1a) in patients affected by a severe form of X-linked intellectual disability originally identified in a multigenerational South African family [1] - since termed Christianson syndrome. The most subtle mutation identified in these patients that would produce a near-intact protein was the two amino acid in-frame deletion ΔE287-S288 (ΔES). These amino acids are highly conserved in all human NHE isoforms (Additional file 1: Figure S1).

To assess the consequences of ΔES deletion on NHE6 biosynthesis, the mutation was engineered into NHE6v1 which contained an HA-epitope at its C-terminus (called NHE6v1_HA_). This construct was then examined in a subline of Chinese hamster ovary cells (AP-1 cells) that expresses negligible levels of endogenous NHE6 [27]. Previous light microscopy analyses indicated that human NHE6 is properly sorted to the plasma membrane and recycling endosomes in this cell line [42]. We now show by electron microscopy that the transporter further partitions to microvilli and other small plasma membrane projections (Additional file 1: Figure S2) present in Chinese hamster ovary cells [47]; a pattern that mimics the trafficking of NHE6 to dendritic spines of hippocampal pyramidal neurons [27]. Thus, these cells provide an ideal tractable model system to study the wild-type (WT) as well as mutant NHE6 transporters in isolation of each other. In addition to AP-1 cells, some experiments were also performed in human neuroblastoma SH-SY5Y cells which do express NHE6 natively [27]. Since NHEs assemble as homodimers [48–50], this would allow us to examine the impact of the mutation on the biosynthesis and post-translational processing of either a homodimer (ΔES/ΔES) or potentially heterodimer (WT/ΔES) in AP-1 and SH-SY5Y cells, respectively. Indeed, biochemical and cellular analyses showed that the WT and ΔES proteins can form a heterodimer protein complex by co-immunoprecipitation assays and partially colocalize in transfected cells (Additional file 1: Figure S3). As presented in Fig. 1b and c and consistent with earlier findings [21, 42], analyses of total cell lysates from AP-1 and SH-SY5Y cells showed that the WT transporter migrated identically in both preparations as multiple bands that reflect the oligomeric and post-translational oligosaccharide state of the transporter: (1) a slower migrating, high molecular weight band (~200–250 kDa) representing the fully-glycosylated dimeric form of the exchanger that does not fully dissociate under SDS-PAGE conditions; (2) a major diffuse, fully-glycosylated form(s) of the monomeric protein (~100–120 kDa); and (3) minor faster migrating, lower molecular weight bands characteristic of the newly synthesized core-glycosylated (~70 kDa) or unglycosylated (~65 kDa) monomers. By contrast, the level of expression of the ΔES mutant was noticeably reduced compared to WT in both cell lines, but especially in AP-1 cells. Densitometric analysis of the immunoblots (using multiple exposures of the immunoblots to ensure the signal intensities of the bands were within the linear range of the X-ray film) indicated that the levels of expression of the ΔES mutant in AP-1 and SH-SY5Y cells were 22.1 % ± 3.2 (*n* = 3) and 79.8 % ± 4.0 (*n* = 3) of WT levels (normalized to GAPDH expression), respectively. Unlike WT, in both cell lines the ΔES mutant migrated predominantly as a single band at the level of the immature core-glycosylated or unglycosylated monomers. However, fainter diffuse bands corresponding to the predicted fully-glycosylated monomer (~100–120 kDa) and/or the fully- (~250 kDa) and core-glycosylated (~175 kDa) dimers were visible, suggesting that a fraction of the mutant protein is capable of undergoing further processing and oligosaccharide maturation. The similar patterns of expression of ΔES in both cell lines also indicate that though the overall abundance of the ΔES mutant was greater in the SH-SY5Y cells, possibly due to stabilizing effects mediated by dimerization with endogenous WT NHE6, the relative oligosaccharide processing of the exogenous ΔES mutant remained compromised.

To confirm the post-translational oligosaccharide state of the proteins, cell lysates of AP-1 cells transiently expressing NHE6v1_HA_ WT or ΔES constructs were prepared using detergent-free buffers and then subjected to treatment with either endoglycosidase H (EndoH), which cleaves only asparagine-linked mannose-rich oligosaccharides (i.e., core-glycosylated) but not more highly processed complex oligosaccharides (i.e., fully-glycosylated), or peptide-N-glycosidase F (PNGaseF) which cleaves between the innermost N-acetylglucosamine and asparagine residues of all oligosaccharide structures (i.e., high mannose, hybrid, and complex). As shown in the immunoblots presented in Fig. 1d and e for WT and ΔES, respectively, EndoH treatment decreased the size of only the core-glycosylated dimeric (~175 → ~165 kDa) and monomeric (~70 → ~65 kDa) proteins for both WT and ΔES, whereas the more diffuse fully-glycosylated dimeric (~250 kDa) and monomeric (~100–120 kDa) bands were unaffected. Conversely, PNGaseF removed essentially all the oligosaccharide moieties for both WT and ΔES, resulting in smaller dimeric and monomeric bands that migrated at ~165 and ~65 kDa, respectively.

To assess the relative contribution of Glu^287^ and Ser^288^ to the observed processing behaviour, we engineered the following point mutations into NHE6v1: the single conservative (i.e., E287Q) and non-conservative (i.e., E287A or S288A) substitutions as well as the double substitution E287Q/S288A. The electrophoretic profiles of the conservative E287Q as well as the S288A mutants appear similar to WT (Additional file 1: Figure S4). By contrast, mutants containing the double E287Q/S288A and single non-conservative E287A substitutions showed reduced abundance, though not as severe as ΔES. Collectively, these data suggest both residues work synergistically to promote the proper maturation of NHE6v1, though Glu^287^ appears more critical.

**Fig. 2:**
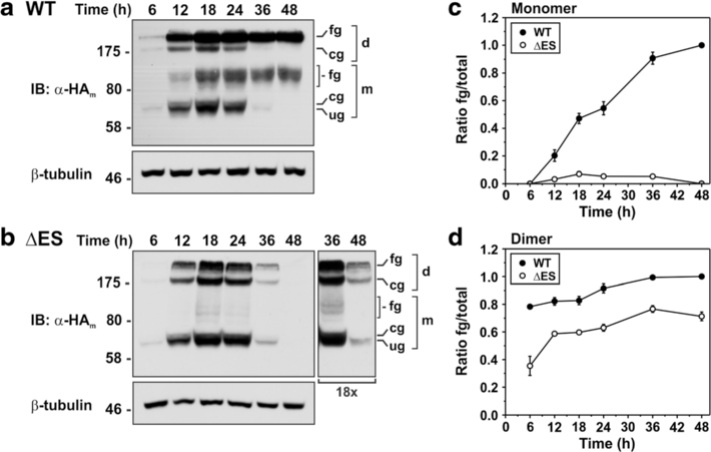
Biosynthetic maturation of NHE6 is reduced for the ∆ES mutant. AP-1 cells were transiently transfected with **a** NHE6v1_HA_ WT or **b** ΔES and lysed at the indicated time points over a 48 h period. Equal amounts of proteins were subjected to SDS-PAGE and immunoblotting with a monoclonal anti-HA antibody (α-HA). The identities of the various NHE6 bands are as described in the legend to Fig. 1. For the ΔES immunoblot in *panel B*, a longer X-ray film exposure (18X) of the 36 h and 48 h time points is also shown. The same immunoblots were also probed with a monoclonal anti-β-tubulin antibody as a loading control. **c-d** Densitometric quantification of the relative abundances of the monomeric and dimeric forms of WT or ΔES was assessed using ImageJ software and expressed as ratios of fully glycosylated/total protein (fg/total). For quantification, multiple exposures of the immunoblots were taken to ensure the signal intensities of the bands were within the linear range of the X-ray film. Data are shown as mean ± standard error of the mean (S.E.M.) of four different experiments

The marked reduction in abundance of the ΔES mutant might be due to impaired biosynthetic maturation, reduced stability, or a combination of the two. To obtain an approximate measure of their biosynthetic maturation, AP-1 cells were transiently transfected with NHE6v1_HA_ WT or ΔES constructs, and cellular lysates were obtained at periodic intervals over 48 h. Quantitative immunoblot analysis revealed that the WT protein is efficiently processed to the fully-glycosylated mature form (monomer and dimer) within 12 h, such that at 36 h most of the transiently synthesized protein is fully processed (Fig. 2a, c). On the contrary, while protein production and dimer assembly of the ΔES mutant are similar to WT during the first 6–12 h, the subsequent post-translational maturation of the protein is deficient, as revealed by a marked reduction in the addition of complex sugars at 18 h which is more apparent in the dissociated monomeric form (Fig. 2b, d). Moreover, the abundance of ΔES is greatly decreased at 36 and 48 h after transfection relative to WT, suggesting that it might be subject to more rapid degradation.

**Fig. 3:**
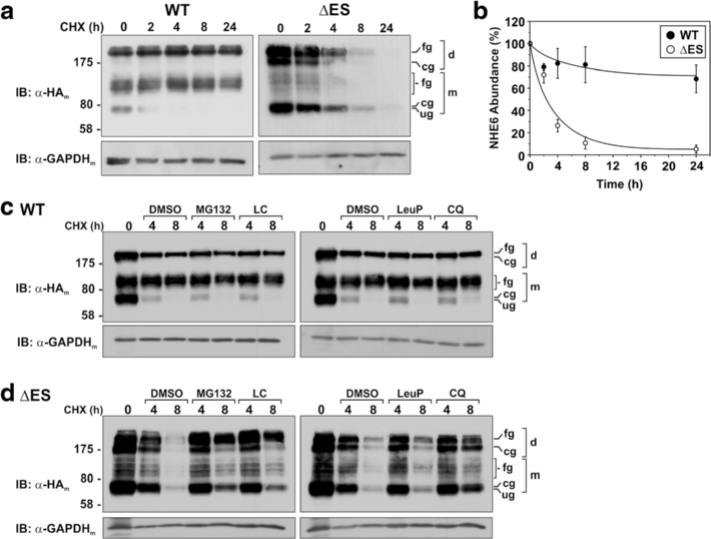
Stability of NHE6 is diminished for ΔES mutant. **a** AP-1 cells were transiently transfected with NHE6v1_HA_ WT or ΔES mutant for 24 h and then treated with 150 μg/mL cycloheximide for the indicated time points, lysed and analysed by SDS-PAGE and immunoblotting with a mouse monoclonal anti-HA (α-HA_m_) antibody. Equal amounts of proteins were loaded, as shown by probing the membranes with a monoclonal anti-GAPDH antibody (α-GAPDH_m_). **b** Quantitative analysis by densitometry of NHE6v1 WT and ΔES protein abundance (normalized to GAPDH levels) as a function of time in the presence of cycloheximide. Values represent the mean ± S.E.M. of three separate experiments. **c-d** AP-1 cells were transiently transfected with NHE6v1_HA_ WT (**c**) or ΔES (**d**) for 24 h and then treated with 150 μg/mL cycloheximide for the indicated time points in the presence of DMSO (vehicle), the proteasomal inhibitors MG-132 (40 μM) or lactacystin (LC, 30 μM) (*left panels*), or the lysosomal inhibitors leupeptin/pepstatin (LeuP, 100 μg/ml) or chloroquine (CQ, 500 μM) (*right panels*). Cellular lysates were analysed by immunoblotting with a mouse monoclonal α-HA_m_ antibody. Membranes were also probed with a mouse monoclonal α-GAPDH_m_ antibody as a loading control

In order to estimate the half-lives of the respective proteins, AP-1 cells were transfected with WT or ΔES for 24 h and then treated with cycloheximide for an additional 2 to 24 h in order to block de novo protein synthesis while tracking the fate of the previously synthesized transporters. As shown in the immunoblots presented in Fig. 3a and quantified in Fig. 3b, the WT protein was relatively stable even after 24 h of treatment (*t*_1/2_ > 24 h). By contrast, the ΔES mutant was rapidly degraded with a calculated half-life of ~2.5 h. As a loading control, the expression of the glycolytic enzyme glyceraldehyde-3-phosphate dehydrogenase (GAPDH) was measured and found to be invariant over the treatment period. The enhanced degradation of the ΔES mutant could arise by shuttling the defective protein to proteasomes and/or lysosomes, pathways previously implicated in the proteolysis of the shorter NHE6v2-ΔES splice variant [43]. Incubating the cells with MG132 or lactacystin, two widely used inhibitors of the proteasomal machinery, concurrently with cycloheximide did not noticeably alter the levels of NHE6 WT over a subsequent 8-h period compared to diluent (dimethylsulfoxide, DMSO) controls (Fig. 3c), whereas they markedly abrogated the decline in the levels of ΔES (Fig. 3d). Similarly, incubating the transfected cells with lysosomal inhibitors leupeptin/pepstatin or chloroquine did not noticeably affect the abundance of the WT transporter, whereas degradation of the ΔES mutant was lessened significantly by either treatment regimen. These results reveal that deletion of amino acids Glu^287^ and Ser^288^ in NHE6v1_HA_ drastically decreases the stability of the protein. Moreover, this degradation appears to be performed by two pathways; initially by the proteasomal machinery indicating that disposal of the mutant protein is mediated by the endoplasmic reticulum-associated degradation (ERAD) pathway [51, 52] and subsequently by the peripheral (i.e., plasma membrane and endosomes) protein quality control machinery that sorts conformationally impaired membrane proteins that escape the ERAD pathway to lysosomes (i.e., endosomal sorting complex required for transport (ESCRT)–dependent lysosomal degradation) [53, 54].

**Fig. 4:**
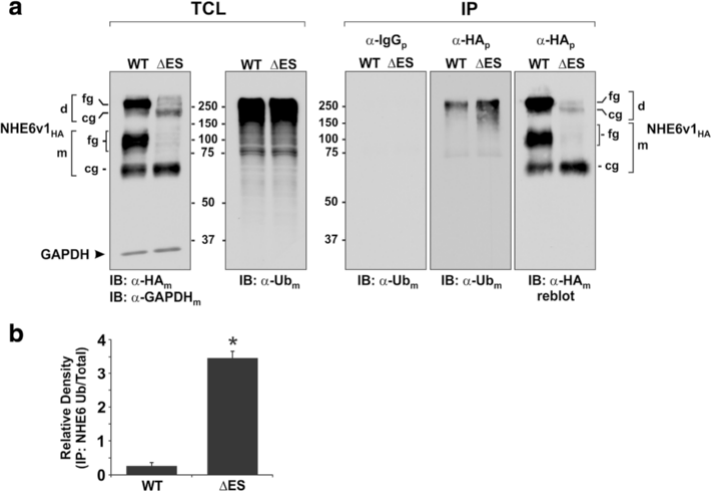
Ubiquitination of NHE6 is increased for the ΔES mutant. **a** AP-1 cells transiently expressing NHE6v1_HA_ WT or ΔES for 24 h were lysed and total levels of NHE6 were analyzed by immunoblotting with a mouse monoclonal anti-HA (α-HA_m_) antibody. GAPDH was measured as a loading control (*panel 1, far left*). The level of ubiquitinated proteins in the total cell lysates (TCL) was examined using a mouse monoclonal anti-ubiquitin (α-Ub_m_) antibody (*panel 2*). The TCL were subjected to immunoprecipitation with a non-specific rabbit polyclonal IgG (α-IgG_p_) antibody (*panel 3*) or a rabbit polyclonal anti-HA (α-HA_p_) antibody (*panel 4*) and analyzed with a mouse monoclonal α-Ub_m_ antibody to detect the ubiquitination levels of NHE6 WT versus ΔES. The membrane from *panel 4* was then stripped and reprobed with a mouse monoclonal α-HA_m_ antibody to examine the total amount of NHE6 WT and ΔES retrieved by immunoprecipitation (*panel 5*). **b** Quantitative analysis by densitometry of the ratio of ubiquitinated to total protein abundance of NHE6v1 WT and ΔES in the immunopreciptated pellets. Values represent the mean ± S.E.M. of three separate experiments. Statistical significance was assessed using a Student’s *t*-test, * *p* < 0.01

A common molecular signature of misfolded proteins targeted for degradation by the ERAD or peripheral quality control machinery is enhanced multi-monoubiquitination or polyubiquitination [52, 55–57]. To test biochemically for increased levels of ubiquitin, NHE6v1_HA_ WT and ΔES were transiently expressed (24 h) in AP-1 cells, followed by immunoprecipitation with a rabbit polyclonal antibody against the HA-epitope (α-HA_p_) and immunoblotting with a monoclonal anti-ubiquitin antibody (α-Ub_m_). As illustrated in Fig. 4a and quantified by densitometry in Fig. 4b, the levels of ubiquitination of ΔES were markedly increased (~13-fold) relative to WT on a protein mass basis, as revealed by stripping the ubiquitin-probed immunoblot and reblotting with a monoclonal anti-HA antibody (α-HA_m_) to detect NHE6v1_HA_. The visibly diffuse signals for ubiquitin represent various levels of ubiquitination of NHE6 monomers and dimers that would increase their molecular weight, extending from ~75 to >250 kDa.

### Cell surface abundance and internalization kinetics of NHE6ΔES are reduced in AP-1 cells

**Fig. 5:**
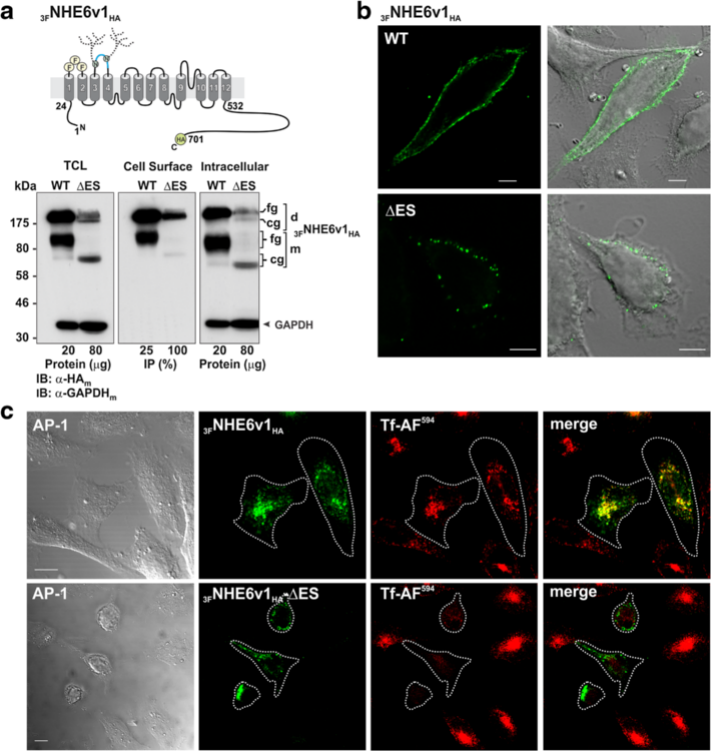
Subcellular distribution of NHE6 ΔES is altered in AP-1 cells. **a** Plasma membrane location of NHE6v1 as measured biochemically using a cell-surface biotinylation assay. To detect cell surface expression of NHE6v1, a triple Flag epitope-tag was inserted into the first predicted extracellular loop of NHE6v1_HA_ (_3F_NHE6v1_HA_), as illustrated in the *upper panel*. AP-1 cells were transiently transfected with _3F_NHE6v1_HA_ WT or ΔES for 36 h and cell surface proteins were labeled with biotin as described in ‘Material and Methods’. Total cell lysates (TCL) were prepared and a small portion representing the total fraction was removed. The remaining supernatants containing equal amounts of total protein for WT and ΔES were loaded onto NeutrAvidin® Agarose beads to purify the biotinylated cell surface proteins from the non-biotinylated (intracellular) proteins. For the TCL and the remaining non-biotinylated fractions, aliquots containing 20 μg and 80 μg protein for WT and ΔES, respectively, were examined by Western blotting (*left and right lower panels, respectively*). For the plasma membrane fraction, 25 % and 100 % of the biotinylated proteins extracted from the total cell lysates of WT and ΔES transfectants, respectively, were subjected to Western blotting (*middle lower panel*). All immunoblots were probed with mouse monoclonal anti-HA_m_ antibody to detect NHE6 and anti-GAPDH_m_ antibody to assess the enrichment of the biotinylated fraction, as GAPDH is a cytosolic protein. **b** Confocal fluorescence microscopy and transmitted light images of fixed non-permeabilized AP-1 cells showing surface expression of _3F_NHE6v1_HA_ WT or ΔES. *Scale bars* represent 5 μM. **c** AP-1 cells were transiently transfected with _3F_NHE6v1_HA_ WT (*upper panels*) or ΔES (*lower panels*). Thirty-six h after transfection, cells were loaded with Alexa Fluor^594^-labelled transferrin (Tf-AF^594^, 10 μg/mL) for 45 min, fixed in 4 % paraformaldehyde, permeabilized, mounted onto glass slides and then examined by confocal microscopy. Footprints of the transfected cells are indicated as white dotted outlines and were derived from the transmitted light images (*far left panels*). *Scale bars* represent 10 μm

Although NHE6 WT accumulates in a perinuclear recycling endosomal compartment, a minor fraction (i.e., ~5-10 % of total NHE6) resides at the plasma membrane as the transporter transits along the recycling endosomal pathway [22, 42]. To investigate whether the ΔES mutant can traffic to the cell surface, plasmalemmal localization was measured biochemically in transfected AP-1 cells using a cell surface biotinylation assay [58]. For these experiments, a triple Flag epitope-tag was inserted in the first extracellular loop of WT and ΔES (_3F_NHE6v1_HA_-WT and _3F_NHE6v1_HA_-ΔES) as illustrated Fig. 5a (*upper panel*). Insertion of epitopes in this position has no discernible effects on the processing, trafficking and function of the transporter [22, 42]. As shown in the immunoblot in Fig. 5a (*lower panel*), the extracted biotinylated cell surface fraction of the WT protein was markedly higher compared to the ΔES mutant, and essentially only fully-glycosylated transporters (intact dimer and dissociated monomer) were detected in the cell surface-enriched fraction. The ΔES mutant protein also reached the cell surface mainly in its dimeric fully-glycosylated form, although a minor amount of the core-glycosylated protein was also detected. The non-biotinylated fractions of WT and ΔES, representative of their intracellular pools that comprise ~90–95 % of total expression, were comparable to their respective abundances in the total cell lysates, as expected. To ensure that the extracted cell surface biotinylated proteins were not contaminated with intracellular proteins, the immunoblots were probed simultaneously with an antibody to GAPDH. GAPDH was readily detected in the total cell lysates and non-biotinylated fractions, but not in the plasmalemmal fractions; thereby confirming the selective enrichment of cell surface proteins. The presence of NHE6v1 WT and ΔES at the plasma membrane was further confirmed visually by imaging of fixed, non-permeabilized AP-1 cells (Fig. 5b). These analyses also showed that the cell surface distribution of NHE6 was discontinuous or punctate. The basis for this is unclear, but may reflect sites of exocytosis and/or endocytosis though other explanations are also possible.

To further characterize the subcellular distribution of NHE6v1 WT and ΔES, dual immunolabelling experiments were performed with various organellar markers using detergent permeabilized cells. As expected, the WT transporter highly co-localized with internalized Alexa Fluor® 594-conjugated transferrin (Tf-AF^594^), a marker of recycling endosomes that is internalized in an adaptor protein 2 (AP2)/clathrin-dependent manner. Conversely, the fluorescence signals for the ΔES mutant showed minimal overlap with Tf-AF^594^ and, furthermore, the intracellular accumulation of Tf-AF^594^ in cells expressing ΔES was visibly reduced relative to neighboring untransfected cells (Fig. 5c). More quantitatively, by calculating the Mander’s overlap coefficient, a statistical parameter that describes the degree of channel overlap that is not dependent upon correlated intensity, the degree of colocalization was significantly reduced for the ∆ES mutant compared to WT (i.e., WT-Tf, 0.80 ± 0.05, versus ∆ES-Tf, 0.36 ± 0.03, *p* < 0.01). Additional subcellular localization analyses showed that immunostaining of ΔES mutant had minimal overlap with signals for the transfected ER marker KDEL_GFP_ [59] and endogenous early endosomal marker EEA1 [60], whereas it overlapped strongly with the transfected late endosomal/multivesicular body marker Rab7_GFP_ [61], suggesting that at 36 h post-transfection the bulk of the defective transporter was being redirected towards the lysosomal degradative pathway (Additional file 1: Figure S5).

**Fig. 6:**
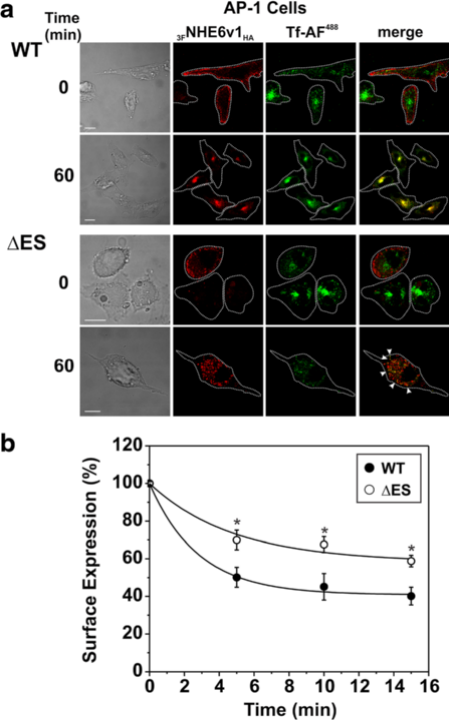
Rate of endocytosis of NHE6 ΔES is reduced in AP-1 cells. **a** AP-1 cells were transiently transfected with _3F_NHE6v1_HA_ WT (*upper panels*) or ΔES (*lower panels*) for 36 h, serum-depleted and then incubated with Alexa Fluor^488^-conjugated transferrin (Tf-AF^488^) for 45 min. Cells were then placed on ice and incubated with primary mouse monoclonal anti-Flag antibody and Alexa Fluor^568^ conjugated secondary antibody, and either fixed in 2 % paraformaldehyde immediately (0 min time point) or after incubation at 37 °C for 60 min. Footprints of the cells within the field of view are indicated as white dotted outlines and were derived from the transmitted light images (*far left panels*). After 60 min of internalization, the signals for the WT transporter highly colocalized with those for Tf-AF^488^, whereas the ΔES mutant showed only partial overlap (the white arrows heads indicate some overlapping punctate signals). Scale bars represent 10 μm. **b** Kinetics of endocytosis measured in AP-1 cells transiently expressing _3F_NHE6v1_HA_ WT or ΔES using a cell-based enzyme-linked immunosorbent assay (ELISA). Thirty-six hours post-transfection, surface NHE6 was labeled on ice using a mouse monoclonal anti-Flag antibody, followed by internalization of the Flag-labeled NHE6 at 37 °C for the indicated time points. Remaining cell surface NHE6 was labeled with a goat anti-mouse HRP-conjugated secondary antibody and detected using the fluorescence Amplex®Red substrate. Data points represent mean ± S.E.M. of four different experiments, each done in triplicate. Statistical significance (*p* < 0.05) was evaluated using a paired two-tailed Student’s *t*-test and indicated by an *asterisk*

Having established that a fraction of _3F_NHE6_HA_ ΔES can reside at the cell surface and intracellular vesicles, we next assessed whether its rate of internalization was different from that of the WT protein. To this end, _3F_NHE6v1_HA_ WT and ΔES were transiently expressed in AP-1 cells and their internalization was examined both visually by confocal microscopy and quantitatively using a cell-based enzyme-linked immunosorbent assay [62]. For image analysis, the cells were preincubated for 45 min with Alexa Fluor® 488-conjugated transferrin (Tf-AF^488^) to label the recycling endosomal compartment, then placed on ice and incubated with primary mouse monoclonal anti-Flag antibody and Alexa Fluor® 568-conjugated goat anti-mouse secondary antibody to label cell surface _3F_NHE6v1_HA_, followed by internalization of the labelled pool at 37 °C for 60 min. Confocal microscopy analysis of these cells revealed the presence of both WT and ΔES at the cell surface before the initiation of endocytosis (Fig. 6a, *first row of upper and lower panels*). After 60 min of internalization, WT was highly concentrated in the perinuclear Tf-AF^488^-containing recycling endosomal pool (Fig. 6a, *upper panel, second row*). By comparison, the punctate signals for ΔES mutant and Tf-AF^488^ were more dispersed throughout the cell after 60 min of internalization rather than coalescing into a more compact perinuclear compartment, but nevertheless were partially overlapping. However, similar to results described in Fig. 5c, the accumulation of Tf-AF^488^ by the ΔES-expressing cells was visibly diminished compared to neighboring non-transfected cells (Fig. 6a, *lower panel, second row*). Quantitative measurements of NHE6 internalization using a cell-based enzyme-linked immunosorbent assay showed that the WT transporter was endocytosed more rapidly than the ΔES mutant (Fig. 6b). Fitting the data to a first order exponential decay function yielded time constants of 2.76 ± 0.48 and 4.81 ± 2.68 min for WT and ΔES, respectively. Collectively, these results indicate that the sequence ^287^Glu-Ser^288^ is important for the efficient biosynthetic maturation and stability of NHE6, which in turn seemingly affects the internalization of NHE6-containing recycling endosomes and associated vesicular cargo.

### NHE6-mediated stimulation of transferrin, but not EGF, uptake is lost in cells expressing the ΔES mutant

**Fig. 7:**
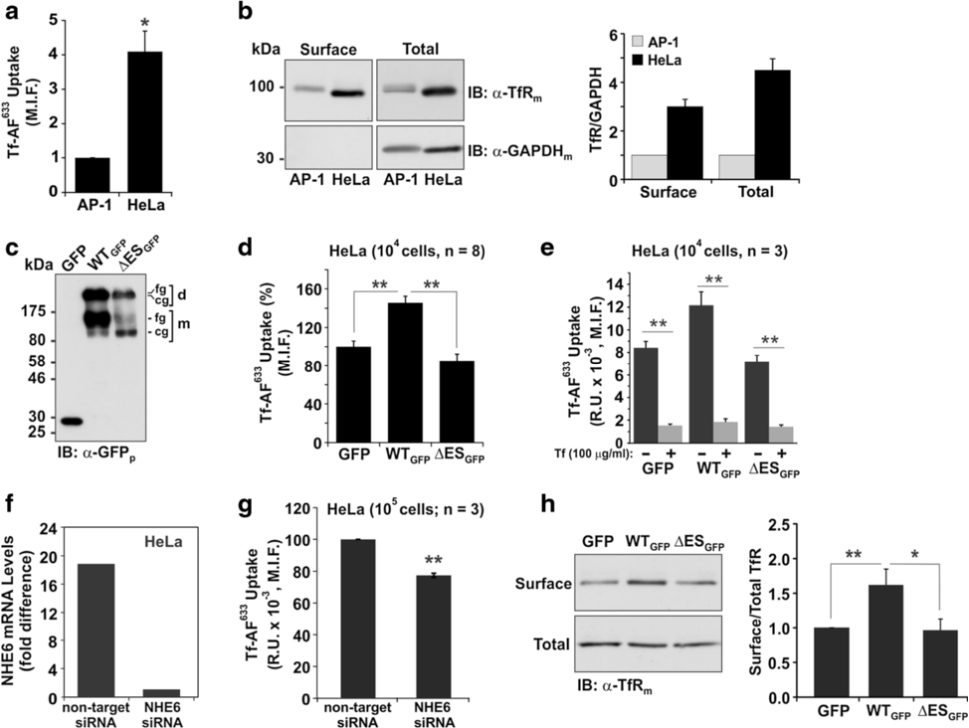
Uptake of transferrin is impaired in HeLa cells expressing NHE6 ΔES. **a** Comparison of uptake of transferrin-Alexa Fluor^633^ (Tf-AF^633^; 10 μg/ml, 5 min) in AP-1 vs. HeLa cells. Significance was measured using a one-sample Student’s *t*-test; *p < 0.05. **b** Surface expression of transferrin receptor (TfR) in AP-1 vs. HeLa cells was measured by cell surface biotinylation. A representative immunoblot shows TfR and GAPDH expression in AP-1 and HeLa cells (*left panel*). Quantification of surface and total TfR relative to total GAPDH expression from three different experiments; values are normalized to the TfR/GAPDH ratio in AP-1 cells (*right panel*). **c** Transient expression of GFP, NHE6v1_GFP_ WT or ΔES in HeLa cells after 48 h. Representative immunoblot probed with a polyclonal anti-GFP (α-GFP_P_) antibody. **d** Uptake of Tf-AF^633^ was monitored in HeLa cells expressing GFP, NHE6v1_GFP_ WT or ΔES. Median fluorescence intensity (M.I.F.) of Tf-AF^633^ was measured in 10^4^ GFP-positive cells by flow cytometry. Data were normalized and represent mean ± S.E.M. of eight different experiments. Significance was established using a one-way ANOVA followed by a Tukey test, **p < 0.01. **e** Uptake of Tf-AF^633^ in HeLa cells expressing GFP, NHE6v1_GFP_ WT or ΔES in the absence (−) or presence (+) of 10-fold excess unlabeled Tf. The M.I.F. of Tf-AF^633^ was measured in 10^4^ GFP-positive cells by flow cytometry. Data represent mean ± S.E.M. of three different experiments, **p < 0.01. **f** qPCR of NHE6 mRNA levels in HeLa cells treated for 72 h with non-target (scrambled) siRNA or NHE6 siRNA. **g** Uptake of Tf-AF^633^ was monitored in HeLa cells expressing non-target siRNA or NHE6 siRNA for 72 h. The M.I.F. of Tf-AF^633^ was measured in 10^5^ cells by flow cytometry. Data were normalized and represent mean ± S.E.M. of three different experiments. Significance was established using a one-sample Student’s *t*-test, **p < 0.01. **h** Cell surface levels of TfR in HeLa cells transiently expressing GFP, NHE6v1_GFP_ WT or ΔES were determined by cell surface biotinylation. Representative immunoblot of TfR expression at the cell surface and the total fraction is shown in the *left panel*. Quantification by densitometry of cell surface/total TfR levels from three different experiments is shown in the *right panel*. Values were normalized to TfR amounts present in GFP-expressing cells and represent the mean ± S.E.M. Significance was established using a one-sample Student *t*-test, ** *p* <0.01, * *p* <0.05

The above imaging results indicated that the uptake of Tf-AF^488^ by ΔES-expressing cells was impaired relative to untransfected neighbouring cells. This observation is consistent with a previous study [25] which reported that siRNA knockdown of NHE6 expression in HeLa cells also decreased internalization of the transferrin receptor (TfR). To determine the effect of the ΔES mutant on internalization of Tf more quantitatively, we developed a flow cytometry-based assay to measure the uptake of red Alexa Fluor® 633-conjugated Tf (Tf-AF^633^). For these experiments, HeLa cells were used instead of AP-1 cells because the signal to noise ratio of Tf-AF^633^ uptake was much greater (4-fold) in HeLa *versus* AP-1 cells at the early linear phase of endocytosis (i.e., 5 min uptake) (Fig. 7a), presumably due to the higher plasma membrane and total cellular abundance (3- to 4-fold) of the TfR in HeLa cells (Fig. 7b). To this end, internalization of Tf-AF^633^ was monitored by flow cytometry in live HeLa cells transiently expressing GFP alone or GFP-tagged constructs of NHE6 WT or ΔES (Fig. 7c). Live cells were distinguished from non-viable cells by their ability to exclude the membrane-impermeant fluorescent dye 7-amino actinomycin D (7-AAD). The median intensity fluorescence (M.I.F.) of internalized Tf-AF^633^ was measured in 10^4^ live (i.e., 7-AAD negative) GFP-positive HeLa cells. As illustrated in Fig. 7d, cells expressing WT_GFP_ exhibited a significant increase in Tf-AF^633^ uptake compared to GFP-only expressing cells (GFP: 100 ± 6 %; WT_GFP_: 145 ± 5 %; *p* < 0.01, one-way ANOVA followed by a Tukey test). However, this stimulation was not evident in cells expressing ΔES_GFP_ and, if anything, showed a slight, albeit statistically non-significant, decrease to 85 ± 7 % (*p* = 0.26) of GFP control levels, suggesting that the mutant is defective in enhancing the uptake of Tf-AF^633^ and possibly other cargo internalized via AP2/clathrin-dependent endocytosis. To validate that the measured fluorescent signal was due to receptor-mediated uptake of Tf-AF^633^ rather than non-specific bulk endocytosis, parallel competition experiments were performed in the presence of 10-fold excess unlabeled Tf. As shown in Fig. 7e, the fluorescent signal was dramatically reduced to background levels in the presence of competing unlabeled Tf, suggesting that the measurements are indicative of receptor-mediated endocytosis of fluorescently-labelled Tf. Moreover, reducing endogenous NHE6 levels in HeLa cells using siRNA (Fig. 7f) decreased Tf-AF^633^ uptake by ~23 % (77 ± 2; *p* < 0.01, one-sample Student’s *t*-test) relative to controls (Fig. 7g). Hence, up- or down-regulation of NHE6 expression reciprocally modulates the endocytosis of the ligand-bound TfR.

Mechanistically, the elevated uptake of Tf-AF^633^ in NHE6_GFP_-WT expressing HeLa cells could arise from an enhanced rate of NHE6-dependent endocytosis due to the elevated abundance of NHE6. However, overexpression of NHE6_GFP_-WT in HeLa cells may also increase the abundance of the TfR at the cell surface due to enhanced recycling or exocytosis of the receptor and thereby increase net Tf-AF^633^ uptake, an effect that is abolished by the ΔES mutation. To examine this latter hypothesis, TfR cell surface levels were examined using a biotinylation assay in HeLa cells expressing GFP, WT_GFP_ or ΔES_GFP_. As shown in Fig. 7h, the levels of TfR at the cell surface were significantly higher (~1.6 fold) in HeLa cells expressing WT_GFP_ as compared to cells expressing GFP alone. By contrast, in cells expressing ΔES_GFP_, no enhanced recruitment of TfR at the plasma membrane was observed. Thus, NHE6 expression levels appear to upregulate the surface abundance and recycling of certain cargo.

**Fig. 8:**
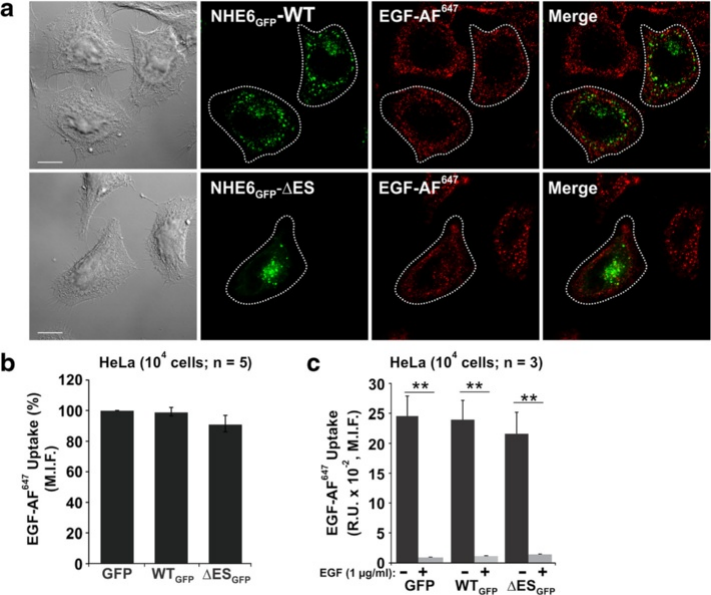
Expression of NHE6 does not affect trafficking of the EGF receptor in HeLa cells. **a** HeLa cells were transiently transfected with NHE6v1_GFP_ WT (*upper panels*) or ΔES (*lower panels*). Forty-eight h after transfection, cells were loaded with Alexa Fluor^647^-labelled EGF (EGF-AF^647^, 100 ng/mL) for 5 min, fixed in 4 % paraformaldehyde, permeabilized, mounted onto glass slides and then examined by confocal microscopy. Footprints of the transfected cells within the field of view are indicated as white dotted outlines and were derived from the transmitted light images (*far left panels*). *Scale bars* represent 10 μm. **b** Uptake of EGF-AF^647^ (100 ng/ml, 5 min) in 10^4^ GFP-positive HeLa cells expressing GFP, NHE6v1_GFP_ WT or ΔES measured by flow cytometry. Data were normalized and represent mean ± S.E.M. of five different experiments. **c** Uptake of EGF-AF^647^ (100 ng/ml, 5 min) in HeLa cells expressing GFP, NHE6v1_GFP_ WT or ΔES in the absence (−) or presence (+) of 10-fold excess unlabeled EGF (1 μg/ml). Median fluorescence intensity (M.I.F.) of EGF-AF^647^ was measured in 10^4^ GFP-positive cells by flow cytometry and values represent the mean ± S.E.M. of three different experiments. Significance was established using a one sample Student *t*-test, **p < 0.001

While NHE6 is seemingly important for the internalization of Tf-TfR, the study by Xinhan et al. [25] also indicated that this effect was somewhat selective, as uptake of other clathrin-mediated cargo such as the epidermal growth factor (EGF) at a concentration of 1 ng/ml was unaffected. However, an earlier study [63] proposed that the EGF receptor (EGFR) could follow different internalization pathways depending on the concentration of its ligand EGF. Accordingly, at low concentrations of EGF (1–2 ng/ml), the EGFR was endocytosed via a clathrin-dependent route, whereas at higher ligand concentrations (10–100 ng/ml), the receptor partitioned roughly equally between clathrin-coated pits and caveolae, suggesting that it can be internalized by both clathrin- and lipid raft-dependent mechanisms [63, 64]. While subsequent studies have disputed these latter findings [65], we nevertheless tested whether NHE6 could internalize the ligand-activated EGFR at higher EGF concentrations that involve not only clathrin mediated endocytosis, but could also potentially favour a caveolae-dependent route, and whether the ΔES mutant impairs this function. To this end, we measured the uptake of EGF-AF^647^ at the higher concentration of 100 ng/ml in HeLa cells. As shown in Fig. 8a, the signals for neither NHE6 WT_GFP_ nor ΔES_GFP_ overlapped with those for the EGFR labelled with EGF-AF^647^ by image analysis. Consistent with this observation, overexpressing WT_GFP_ or ΔES_GFP_ did not alter the uptake of EGF-AF^647^ when compared to control cells expressing GFP alone (Fig. 8b). The specificity of the EGF fluorescent signal was validated by competition experiments in the presence of 10-fold excess unlabeled EGF (Fig. 8c). Thus, even at high concentrations of EGF, NHE6 does not colocalize with the EGF-EGFR complex. These data are consistent with earlier findings by Xinhan et al. [25] and confirm that NHE6 regulates the internalization of some, but not all, clathrin (or potentially caveolin)-dependent cargo, at least in HeLa cells.

### The pH of NHE6ΔES-containing endosomes fails to alkalinize

**Fig. 9:**
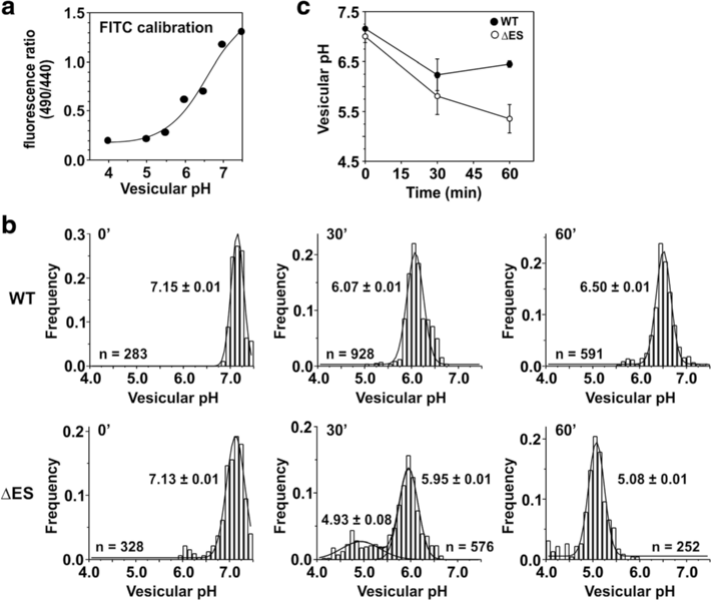
Over-acidification of endosomes containing NHE6 ΔES. AP-1 cells were transiently transfected with _3F_NHE6_HA_ WT and ΔES and endosomal delivery was assessed 36 h post-transfection. Anti-Flag M2 primary and FITC-conjugated Fab secondary antibodies were bound to live cells for 1 h on ice. The temperature was raised to 37 °C for 30 or 60 min and endosomal pH was measured by fluorescence ratio imaging (FRIA). **a** *In situ* calibration of FITC fluorescence as a function of vesicular pH was performed by clamping the vesicular pH between 4 and 7.5 as described in “Methods”. **b** Measurement of vesicular pH as a function of time upon internalization of _3F_NHE6_HA_ WT and ΔES. The pH values represent the mean ± S.E.M. and the number of vesicles (~250 to 1000 vesicles) analyzed from a representative experiment are shown. **c** Graphical plot of the median vesicular pH as a function of time derived from four experiments (mean ± S.E.M.)

The impaired uptake of Tf-AF^633^ in cells expressing NHE6ΔES suggested that recycling endosomal function has been compromised. Since proper acidification of organelles is an important determinant of membrane trafficking [66–68] and because NHE6 is believed to operate as an alkalinizing mechanism to counter the acidification generated by the vacuolar H^+^-ATPase [25], we measured the vesicular pH (pH_v_) of NHE6 WT- and ΔES-containing endosomes by fluorescence ratiometric image analysis (FRIA) [69]. To this end, AP-1 cells were transfected with _3F_NHE6v1_HA_ WT or ΔES. Thirty-six h post-transfection, cell surface NHE6 molecules were labeled on ice with a primary mouse monoclonal anti-Flag antibody, followed by incubation with a Fab secondary antibody conjugated to the pH-sensitive ratiometric dye fluorescein isothiocyanate (FITC). Cells were then incubated in cell culture media at 37 °C for 30 min or 60 min and individual vesicles were analyzed by FRIA. The pH calibration curve is shown in Fig. 9a and an example of the pH distribution of newly formed recycling endosomes as a function of time is presented in Fig. 9b, with the median vesicular pH values from multiple experiments summarized in Fig. 9c. After 30 min of internalization, both WT and ΔES were predominantly targeted to a compartment with median pH_v_ values of ~6.25 ± 0.35 and 5.80 ± 0.39, respectively, consistent with accumulation in early/sorting endosomes, though in the case of ΔES-expressing cells a minor fraction of the transporter was also detected in more acidic vesicles (pH_v_ ~5.0) (Fig. 9b). At 60 min after internalization, the WT protein was found in a more alkaline vesicular pool (median pH_v_ ~6.50 ± 0.09), corresponding to the recycling endosomal compartment, whereas the bulk of the ΔES mutant protein accumulated in a very acidic compartment (pH_v_ ~ 5.37 ± 0.25) (Fig. 9c) more consistent with late endosomes and lysosomes, suggesting that its’ alkalinizing function was compromised. As a control, we also measured the luminal pH of recycling endosomes in untransfected AP-1 cells using FITC-conjugated Tf as a pH-sensitive probe. After 30 min of internalization, the pH_v_ was 6.26 ± 0.14 (mean ± S.E.M., *n* = 4) and remained at that level after 60 min (Additional file 1: Figure S6). These values are intermediate between those obtained for the WT- and ΔES-transfected cells. Collectively, these data further confirm an important role for NHE6 in the late-phase alkalinization of recycling endosomes.

### NHE6 ΔES expression enhances apoptosis in AP-1 cells

**Fig. 10:**
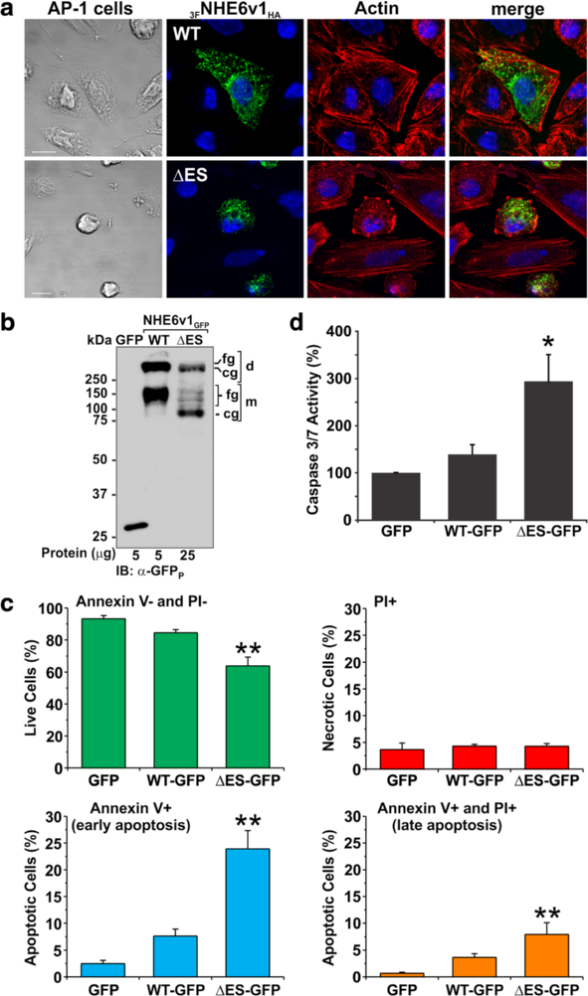
Expression of NHE6 ΔES enhances apoptosis in AP-1 cells. **a** Confocal microscopy of fixed AP-1 cells expressing _3F_NHE6v1_HA_ WT (*upper panels*) or ΔES (*lower panels*). NHE6 was labelled with a mouse monoclonal anti-HA_m_ antibody and an Alexa Fluor^488^-conjugated goat anti-mouse secondary antibody. Actin filaments were labelled with rhodamine-phalloidin and the nuclei were stained with DAPI. **b** Transient expression (48 h) of GFP, NHE6v1_GFP_ WT or ΔES in AP-1 cells. Representative immunoblot probed with a polyclonal anti-GFP antibody (α-GFP_P_). **c** Flow cytometry analysis of AP-1 cells transfected with GFP alone, NHE6_GFP_ WT or ΔES. Forty-eight hours after transfection, cells were labeled with Annexin V-APC and propidium iodide (PI) and 10^4^ GFP-positive cells were examined by flow cytometry for each transfectant. Annexin V- and PI- double negative cells represent viable cells. Cells taking up only PI (PI+) are indicative of dead cells; Annexin V+ positive cells indicate early apoptotic cells whereas Annexin V+ and PI+ double positive cells represent late apoptotic cells. Results are shown as mean ± S.E.M. of eight independent experiments. Significance was determined using a paired two-tailed Student *t*-test, **p < 0.01. **d** AP-1 cells transiently expressing GFP alone, NHE6_GFP_ WT or ΔES were isolated by cell sorting and then assayed for caspase 3/7 activity as described in “Materials and Methods”. Data were normalized to values for GFP-expressing cells and displayed as mean ± S.E.M. of four independent experiments, each done in triplicate. Significance was determined using a paired two-tailed Student *t*-test, *p < 0.05

During the course of these studies, we noted that the morphology of a signficant proportion of AP-1 cells expressing the ΔES mutant appeared smaller, more rounded, showed surface blebbing and exhibited extensive loss of filamentous actin stress fibers (Fig. 10a, Additional file 1: Figure S5), all features consistent with cells undergoing apoptosis [70–72]. To examine this possibility, AP-1 cells were transfected with GFP as a control, GFP-tagged NHE6v1 WT or ΔES (Fig. 10b). Forty-eight hours post-transfection, the cells were incubated in the presence of the fluorescent annexin V-allophycocyanin conjugate (Annexin V-APC) and propidium iodide (PI) and analyzed by flow cytometry to determine the fraction of apoptotic cells. Annexin V is a Ca^2+^-dependent phospholipid-binding protein with high affinity for phosphatidylserine which is normally present in the inner leaflet of the plasma membrane [73]. Propidium iodide is a fluorescent molecule whose signal is enhanced 20- to 30-fold upon binding to double-stranded DNA and RNA but generally cannot cross the intact plasma membrane of viable cells. During the early stages of apoptosis, phosphatidylserine is translocated to the outer leaflet of the plasma membrane, where it is now accessible for binding to an Annexin V-APC probe. However, the integrity of the plasma membrane at this stage is maintained, so PI cannot penetrate inside the cells. As such, Annexin V-APC positive and PI negative cells are considered to be early apoptotic. In the later stages of apoptosis, as well as in necrosis (unregulated cell death) or necroptosis (programmed necrosis) [74], the plasma membrane becomes leaky, allowing PI to enter the cells and to bind to nucleic acids, so Annexin V-APC/PI-double positive cells are late apoptotic or necrotic, whereas PI-only positive cells are considered necrotic [75]. GFP-positive cells (10^4^ cells per experiment) were analyzed and a significantly higher proportion of early and late apoptotic cells were detected among the ΔES-expressing cells compared to cells expressing wild-type NHE6_GFP_ (Fig. 10c). To further substantiate the activation of an apoptotic pathway, which is defined as a caspase-dependent form of regulated cell death [76, 77], we measured caspase 3 and 7 activity using the luminescent Caspase-Glo® 3/7 assay from Promega. To this end, GFP-positive AP-1 cells transiently expressing GFP, NHE6_GFP_ WT or ΔES were isolated by fluorescence-activated cell sorting (FACS) 24 h post-transfection and grown for an additional 24 h. As shown in Fig. 10d, the activation of caspase 3 and 7 is significantly higher (~3-fold) in cells expressing the NHE6_GFP_-ΔES mutant compared to GFP or NHE6_GFP_-WT, consistent with the flow cytometry analyses.

### The distribution of the NHE6ΔES mutant is altered in primary mouse hippocampal neurons

**Fig. 11:**
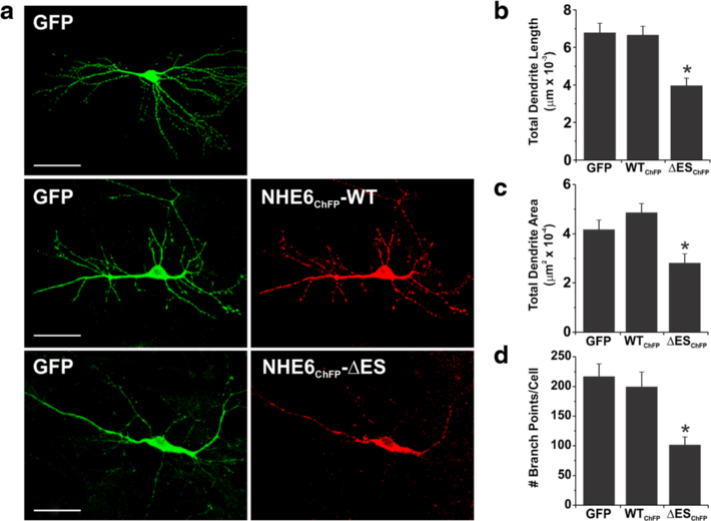
Expression of NHE6∆ES decreases the complexity of dendritic arborisation in primary hippocampal neurons. **a** Confocal images of mouse primary hippocampal neurons transfected with cytosolic GFP alone or co-transfected with GFP and monomeric cherry fluorescent protein-tagged NHE6 (NHE6_ChFP_) WT or ∆ES. Forty-eight h post-transfection, cells were fixed in 4 % paraformaldehyde, mounted onto glass slides, and examined by confocal microscopy. Images show each channel individually. **b-d** Quantification of parameters related to neuronal branching, including the sums of total dendritic length (**b**) and area (**c**), as well as the total number of branch points per cell (**d**), using the FilamentTracer plug-in module from Imaris Software. Transfection of NHE6_ChFP_-∆ES appeared to reduce the extent of neuronal arborisation, as can be discerned from the representative images in **a**. Data from four experiments is represented as mean ± S.E.M. values. *: *p* < 0.01, one-way ANOVA with Tukey post-hoc test. Scale bar: 60 μm

The above results show that NHE6ΔES is mislocalized in non-neuronal cells. To investigate whether the subcellular distribution of the mutant protein is similarly altered in neurons, primary cultures of differentiated hippocampal pyramidal neurons (10–12 days in vitro, DIV) prepared from C57BL/6 mice were transfected with GFP alone or GFP and either fluorescent mCherry (ChFP)-tagged constructs of NHE6 WT or ΔES (NHE6_ChFP_-WT and NHE6_ChFP_-∆ES, respectively) and visualized by confocal microscopy after 48 h. Following fixation and mounting, multiple z-stack optical sections of 300–500 nm were taken and frame averaged 3X at low resolution or line-averaged 2X at high resolution to improve the signal-to-noise ratio. Images were then deconvolved by using a full maximum likelihood extrapolation algorithm Huygens deconvolution software (SVI), and 3D images were compiled as maximum intensity projections using Imaris software (Bitplane AG). As shown in Fig. 11a, representative neurons expressing control GFP alone or GFP and NHE6_ChFP_-WT exhibited extensive dendritic arborization, whereas those expressing GFP and NHE6_ChFP_-∆ES displayed an apparent reduction in higher-order dendritic branching, though the number of primary dendrites (originating from the soma) was similar to controls (GFP: 5.67 ± 0.95; WT: 4.86 ± 0.59; ∆ES: 4.14 ± 0.44; *p* > 0.05). Using FilamentTracer Imaris software, there were significant reductions in total dendritic length, surface area and number of branch points of the neurons expressing NHE6_ChFP_-∆ES (~50 % for each parameter; *p* < 0.01 one-way ANOVA followed by a Tukey test) compared to control GFP or GFP and NHE6_ChFP_-WT (Fig. 11b-d).

**Fig. 12:**
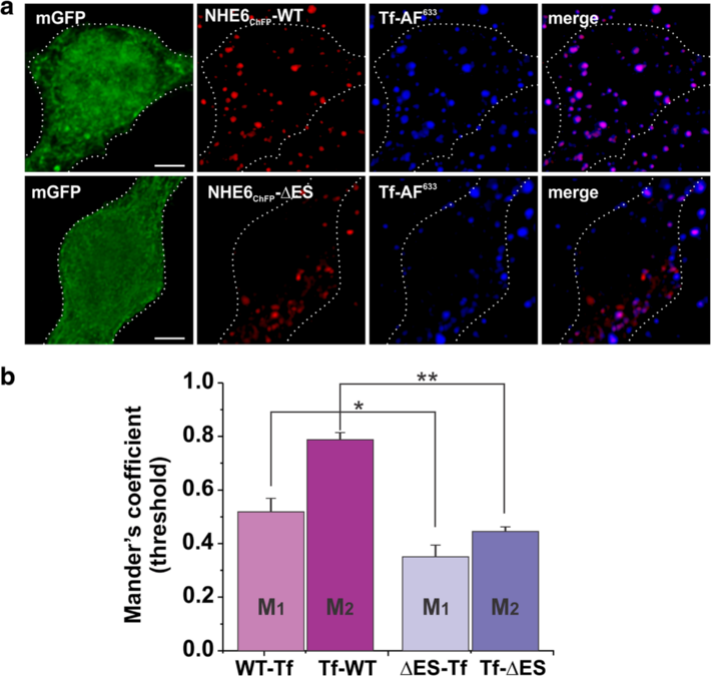
Expression of NHE6∆ES causes a reduction in transferrin uptake and colocalization in primary hippocampal neurons. **a** Confocal images of the cell bodies of primary hippocampal neurons transfected with GFP (*green*) and NHE6_ChFP_ WT or ∆ES (*red*) and incubated with Alexa Fluor^633^-tagged transferrin (Tf-AF^633^) (pseudo-coloured blue) to assess endocytotic transferrin uptake and colocalization. Images show each channel individually, with merged images of the NHE6_ChFP_ and Tf-AF^633^ channels. **b** Quantitative summary of mean ± S.E.M. thresholded Mander’s coefficients, a measure of colocalization, between Tf-AF^633^ and NHE6_ChFP_ from four experiments. By the Mander’s coefficient, the majority of Tf-AF^633^ was colocalized with NHE6_ChFP_ WT than the inverse. The degree of colocalization was decreased with the NHE6_ChFP_ ∆ES mutant. *: *p* < 0.0001; **: *p* < 0.05, independent Student’s *t*-test, two-tailed. Scale bar: 10 μm

To investigate the vesicular nature of the NHE6-positive puncta, dual labelling experiments were performed using Tf-AF^633^ to mark recycling endosomes. Visual analysis revealed that within the soma, the signals for NHE6 WT closely overlapped with those for Tf-AF^633^, though this spatial relationship diminished markedly for the ∆ES mutant (Fig. 12a). Quantitative calculation of the Mander’s overlap coefficient M1-M2 indicated that the degree of colocalization was significantly reduced for the ∆ES mutant compared to WT (i.e., M1; WT-Tf, 51.9 % ± 5.1, versus ∆ES-Tf, 35.0 % ± 4.4, *p* < 0.05; M2; Tf:WT, 78.7 % ± 2.7 versus Tf-∆ES, 44.6 % ± 1.7, *p* < 0.01) (Fig. 12b). These data suggest that the ∆ES mutant is being partitioned away from the recycling endosomal pool, results consistent with those obtained for AP-1 cells.

**Fig. 13:**
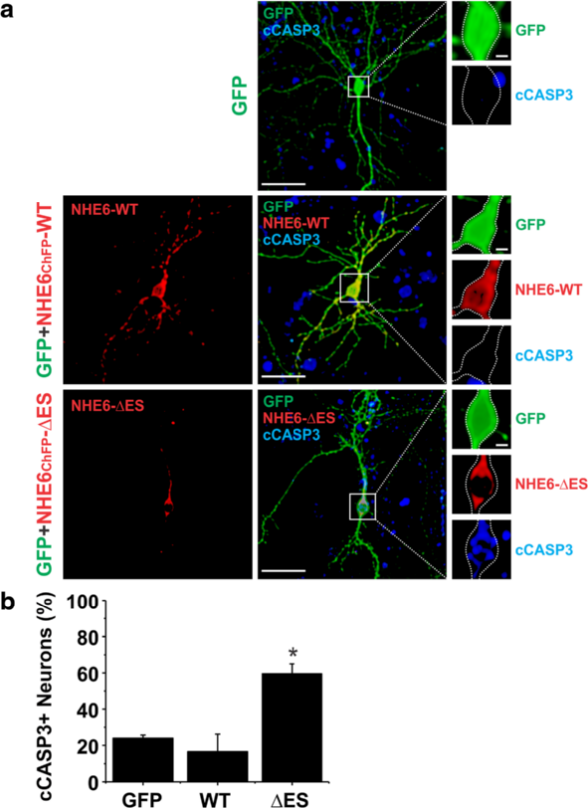
Expression of NHE6∆ES induces apoptotic cell death of primary hippocampal neurons. **a** Representative confocal images of primary hippocampal neurons transfected with cytosolic GFP alone or GFP co-transfected with either NHE6_ChFP_-WT or NHE6_ChFP_-∆ES. Forty-eight h post-transfection, cells were fixed, permeabilized, blocked, and assessed for apoptosis by immunostaining for cleaved caspase-3 (cCASP3, *blue*). For each transfection condition, an overview is presented of the entire transfected neuron with the GFP, ChFP, and cCASP3 channels merged (*middle panels*) with higher magnification cut-away images of the area around the cell soma (indicated by the *white* square) with each channel displayed separately (*right panels*). For the co-transfected cells, the signals for the ChFP-tagged NHE6 constructs are also shown separately (*left panels*). As noted in the images, cCASP3 was also detected in nontransfected cells, which could include not only neurons, but also astrocytes and glia. Hence, to estimate the background level of apoptotic cells per field of view, companion cultures in each preparation were fixed, permeabilized and immunolabelled for cCASP3 and stained with propidium iodide to mark the nuclei in order to calculate total cell density/field of view. Under each treatment condition, the average number of cells per field of view ranged from 350 to 400 and the percentage of apoptotic cells per field of view for GFP, GFP + NHE6_ChFP_-WT or GFP + NHE6_ChFP_-∆ES was 9.8 % ± 0.7, 9.7 % ± 1.0 and 9.1 % ± 0.8 (mean ± S.E.M.), respectively. Hence, ~10 % of the cells in the background were apoptotic under each condition. **b** Quantitative representation of the percentages of cCASP3-positive (cCASP3+) neurons of examined GFP or GFP- and NHE6_ChFP_ transfected cells within each condition for four separate experiments. Data are presented as the mean ± S.E.M. (total transfected cells examined ranged from 21–25 cells per condition). Compared to transfection with GFP alone or GFP + NHE6_ChFP_-WT, significantly more neurons transfected with GFP + NHE6_ChFP_-∆ES were cCASP3-positive. *: *p* < 0.01, one-way ANOVA with Tukey post-hoc test. Scale bar: whole cell images, 60 μm; high magnification images, 10 μm

Given the above observations, we next investigated the viability of the primary neurons transiently expressing GFP alone, NHE6_ChFP_-WT or NHE6_ChFP_-∆ES by immunolabelling for the activated, cleaved form of caspase-3 (caspase-3 (Asp175); cCASP3) as a convenient indicator of apoptotic cell death. As shown in Fig. 13, the percentage of neurons expressing GFP alone or GFP and NHE6_ChFP_-WT that co-stained for cCASP3 was low in each case (~ < 20 %), whereas the percentage of transfected cells expressing NHE6_ChFP_-∆ES that were positive for cCASP3 increased 3-fold, indicative of pronounced cell death.

## Discussion

Patients with CS possess a spectrum of core and secondary clinical symptoms [1, 3, 8, 9], the variability of which may depend on the severity of the mutation in NHE6, functional overlap with other pH-regulating solute carrier proteins, or genetic-modifier effects. In the present study, we examined in detail the molecular and cellular consequences of one of the originally identified CS mutations that causes an in-frame deletion of two highly conserved amino acids in the predicted seventh transmembrane helix of the transporter, using the longest splice-variant as a template (i.e., NHE6v1Δ^287^ES^288^). Compared to the WT protein, excision of these two residues markedly reduced its rate of post-translational maturation to complex oligosaccharides, as revealed by a greater accumulation of its core-glycosylated form relative to the total ΔES population in transient transfection assays in both fibroblastic and neuroblastoma cell lines. This correlated with a significant reduction (~10-fold) in its half-life compared to WT, effects that could be partially prevented by inhibitors of both proteasomal- and lysosomal-mediated proteolysis; indicative of processing via the endoplasmic reticulum (i.e., ERAD) and peripheral (i.e., ESCRT) quality control pathways, respectively. Activation of ERAD indicates that newly synthesized mutant proteins have an increased propensity to misfold and undergo multi-monoubiquitination or polyubiquitination and retrotranslocation from the ER to the cytoplasm for degradation by the proteasome [51, 52]. Consistent with this notion, the overall state of ubiquitination of the ΔES mutant was significantly elevated (~13-fold) compared to WT. By light microscopy, there was limited accumulation of the mutant protein in the ER, suggesting that the ERAD system was not overly saturated due to ectopic expression under our experimental conditions and could adequately handle the misfolded mutated proteins. The remaining fraction of mutated NHE6, however, was able to undergo posttranslational modification and assembly as a fully-glycosylated homodimer and, furthermore, was delivered correctly to the plasma membrane. However, upon internalization, the ΔES mutant was redirected away from the recycling endosomal compartment as it showed minor overlap with markers of recycling endosomes (transferrin) and early endosomes (i.e., EEA1), and instead accumulated in Rab7-associated late endosomes/multivesicular bodies prior to degradation in lysosomes. Consistent with the microscopic evidence, degradation of this population of transporters could be partly attenuated by lysosomal inhibitors. These findings are comparable to the lysosomal degradation of other misfolded plasma membrane proteins that escape the ERAD pathway, such as certain mutant Cl^−^ channels (i.e., cystic fibrosis transmembrane regulator, CFTR) responsible for cystic fibrosis [78, 79] and defective K^+^ channels (i.e., human ether-a-go-go-related, hERG) that cause long QT syndrome 2 [80], and highlight the importance of a secondary peripheral quality control mechanism to eliminate the accumulation of improperly folded proteins. These findings partially corroborate those of an earlier study that showed that the analogous mutation in the shorter NHE6v2 splice-variant (i.e., Δ^255^ES^256^) also caused the protein to be highly unstable and rapidly degraded by the proteasome and lysosome [43]. However, in this latter study, the GFP-tagged WT and Δ^255^ES^256^ proteins were detected as single ~70 kDa bands by Western blotting which is considerably smaller than its predicted molecular mass of ~100 kDa, and unlike the multiple core- and fully-glycosylated monomers and dimers detected in the present study using the longer NHE6v1 variant. Moreover, the GFP-tagged NHE6v2-Δ^255^ES^256^ protein accumulated mainly in the endoplasmic reticulum, with only a minor fraction detected in early endosomes but not recycling endosomes. The basis for these differences is unclear, but may relate to technical variances or the biochemical natures of the respective splice-variants.

Aside from the intrinsic instability of the NHE6v1-ΔES protein, its rate of internalization from the cell surface of AP-1 cells was also significantly reduced compared to its WT counterpart. By microscopy, ΔES-expressing AP-1 cells showed visibly reduced uptake and accumulation of fluorescently-labelled Tf compared to neighboring untransfected cells. Comparable results were also obtained in HeLa cells using a flow cytometry-based assay where the net uptake of the labelled Tf-TfR complex was significantly lower in ΔES compared to WT transfected cells, and slightly lower that values obtained for GFP-transfected control cells, though the latter difference was not statistically significant. One possibility for the apparent lack of a strong dominant-negative effect of the ΔES mutant in HeLa cells compared to AP-1 cells is that its level of expression might not be sufficient to completely suppress the actions of the endogenous NHE6 WT transporter, which is well expressed in HeLa cells [23] but negligible in AP-1 cells. For this reason, we also performed a siRNA knockdown (>95 %) of NHE6 in HeLa cells to validate its involvement in endocytosis of Tf-TfR complexes, and indeed we observed a small, but statistically significant, depressive effect (~23 %, Fig. 7g), consistent with earlier findings [25]. By contrast, other clathrin-mediated cargo such as the EGF-bound EGFR was unaffected. The molecular basis for the differential regulation of clathrin-dependent cargo by NHE6 is unknown, but may relate to the recruitment of different endocytic adaptor proteins and associated accessory proteins. Unlike the activated Tf-TfR which is highly dependent on the AP2 adaptor complex for internalization [81], the EGF-EGFR is less restricted and can bind to AP2 as well as alternate endocytic adaptors such as epsin-1 [82] and Grb2 [83, 84] and then is preferentially sorted to the lysosome for degradation. Hence, NHE6 appears to play a role in the endocytosis of a discrete subpopulation of clathrin-dependent cargo that is preferentially targeted to recycling endosomes in HeLa cells and presumably other cell types as well. In addition, we found that overexpression of NHE6 WT, but not ΔES, also increased the abundance of TfR at the plasma membrane. Based on these data, we propose that NHE6 elevates the net uptake of Tf not only by enhancing endocytosis but also in part by promoting the exocytosis and steady-state cell surface abundance of the TfR, and that this upregulation is deficient in cells expressing the ΔES mutant.

The deficit in Tf uptake in ΔES-expressing cells also correlated with aberrant over-acidification of endosomes relative to those in WT-expressing cells. Using an immunological-based approach that selectively targeted a pH-sensitive fluorescent probe to the lumen of NHE6-containing vesicles in AP-1 cells, we found that WT-containing vesicles initially acidified (i.e., pH_v_ ~6.25 ± 0.35) followed by a gradual alkalization (i.e., pH_v_ ~6.50 ± 0.09) over a 60 min period. This biphasic pH fluctuation is consistent with previous reports of pH transients along the recycling endosomal pathway [85–87]. By contrast, the ΔES-containing vesicles became progressively more acidic throughout the measurement period (i.e., pH_v_ ~ 5.37 ± 0.25). This suggests that the catalytic activity of the mutant was compromised and unable to counter the H^+^ influx driven by the vacuolar H^+^-ATPase, resulting in a net increase in the luminal H^+^ concentration. This loss-of-function is perhaps not unexpected as mutation of the analogous glutamate residue (i.e., E262) in the plasmalemmal-type NHE1 isoform was also found to significantly decrease its total cellular and plasma membrane abundance as well as intrinsic catalytic activity (~20 % of wild-type activity) [88]. Thus, this glutamate residue is critical not only for protein stability, but also for its catalytic activity. The increased endosomal acidification would also be consistent with the partitioning of ΔES-containing vesicles and any associated cargo towards the endo-lysosomal degradative pathway.

Acidification has long been recognized as an important determinant of vesicular biogenesis, trafficking and function [67, 86, 87, 89]. While the roles of acidification on enzymatic processing of proteins along the secretory and degradative pathways are well appreciated, the precise mechanisms by which intraorganellar pH is sensed and transmitted to the cytoplasmic molecular machinery that controls vesicular events such as budding, coat formation, sorting and fusion are less well understood. However, emerging evidence indicates that endosomal pH-regulators themselves can serve as both pH-sensors and scaffolds to recruit components of the vesicular trafficking machinery. Recent studies by Marshansky and colleagues [90, 91] have shown that two distinct subunits of the transmembrane V_0_ complex of the vacuolar H^+^-ATPase, the c- and a2-subunits, directly recruit the small GTPase Arf6 (ADP-ribosylation factor 6) and its associated guanine nucleotide exchange factor ARNO (ADP-ribosylation factor nucleotide site opener), respectively, in an intra-endosomal pH-dependent manner; interactions that are critical for endosomal trafficking between the early and late endosomal compartments. This process is seemingly selective, as it does not appear to influence membrane trafficking along the recycling endosomal pathway. This is intriguing, but unexpected, since previous findings had also linked Arf6 to the recycling pathway [92–94]. This suggests that other endosomal pH-regulatory transporters, such as NHE6, may play a more prominent role in directing vesicular trafficking along the recycling endosomal pathway and that this process is impaired in the ΔES-expressing cells.

In addition to disrupting recycling endosomal pH and trafficking, we found that expression of the NHE6 ΔES mutant in AP-1 cells elicited morphological and biochemical changes symptomatic of programmed’apoptotic’ cell death [95, 96], as revealed by (1) disassembly of the filamentous actin network accompanied by cell rounding and retraction, (2) plasma membrane blebbing, phospholipid flipping (i.e., external exposure of phosphatidylserine) and permeabilization, and (3) elevated activities of caspases 3 and 7. Similarly, ectopic expression of ΔES in primary hippocampal neurons showed aberrant subcellular distribution of ΔES-containing endosomes and pronounced neurodegeneration, as manifested by significantly reduced dendritic length, surface area and number of secondary branch points as well as increased activation of caspase 3; features consistent with regulated cell death [97]. These observations complement earlier in vitro studies showing that manipulations that disrupt the molecular machinery involved in recycling endosomal trafficking at dendritic spines of hippocampal neurons also cause pronounced morphological changes, including decreased dendritic spine size and density and impair long-term potentiation [98, 99]. Indeed, we have recently shown that NHE6 exhibits a high degree of colocalization with vesicles containing the glutamatergic AMPA GluA1-containing receptor in dendrites and dendritic spines of hippocampal neurons, suggestive of a role for NHE6-containing vesicles in synapse formation, maturation, and plasticity [27].

The above findings are also consistent with in vivo observations of progressive neurodegeneration in NHE6 null mice [26, 28] and Christianson syndrome patients [1, 3, 8, 9]. In the null mouse model, both mutant male (*Nhe6*^-*/Y*^) and homozygous female (*Nhe6*^*−/−*^) knockout mice exhibit impaired endo-lysosomal function (i.e., aberrant accumulation of GM2 ganglioside and cholesterol) in subpopulations of neurons within the amygdala, hippocampus, hypothalamus and cerebral cortex as well as pronounced formation of axonal spheroids and degeneration of cerebellar Purkinje cells, features typical of many lysosomal storage diseases [26, 100]. Furthermore, hippocampal and cortical pyramidal neurons of *Nhe6* knockout mice examined in vivo and in vitro displayed morphological and functional abnormalities typified by enhanced endosomal acidification, reduced axonal and dendritic arborization, decreased synapse density and maturation, and impaired circuit activity [28]. These changes correlated with marked decreases in the levels of total and phospho-activated forms of the neurotrophin receptor TrkB, effects that could be largely mitigated by pharmacological inhibitors of lysosomal proteolysis or by chronic incubation with the exogenous TrkB ligand BDNF [28]. Moreover, immunohistochemical staining revealed substantial colocalization of NHE6 and TrkB in endosomes in the perinuclear region and along growing axons and dendrites of hippocampal neurons. Thus, loss of NHE6 was proposed to lead to excess degradation of the TrkB receptor (and possibly neurotransmitter receptors such as AMPAR) and attenuation of downstream signalling due to over-acidification of the endosome compartment and sorting to lysosomes. Impairment in TrkB signaling has also been implicated in the development of Angelman Syndrome [101], a disorder that bears many features in common with Christianson Syndrome [8]. Hence, these findings suggest that disruption of endosomal trafficking that promotes neurotrophin receptor-mediated prosurvival signals (e.g., via TrkB) [31, 102–104] may shift the balance towards neurotrophin receptor-mediated proapoptotic signals (e.g., via p75^NTR^) [105–108] leading to neuronal cell death. Analogous perturbations of plasma membrane/endomembrane-triggered signalling pathways may also apply in non-neuronal cells expressing the NHE6-ΔES mutant when the equilibrium between prosurvival growth factor receptors and proapoptotic death- or dependence-receptors is chronically perturbed [109–112].

While loss of NHE6 function may disrupt trophic or activity-dependent survival signals leading to cell deterioration and death, ER stress [113] may also be another important factor that reduces cell function and viability in cells possessing the ΔES mutant. The mutant protein might trigger the unfolded protein response (UPR), an intricate homeostatic process that results in the arrest of general protein translation, while simultaneously permitting enhanced production of molecular chaperones involved in protein folding, and increased protein polyubiquitination and export of misfolded proteins to the cytoplasm for proteasome-mediated degradation [114, 115]. Consistent with this process, the ΔES mutant is highly ubiquitinated and its degradation can be partly blocked by proteasomal inhibitors. However, like many neurodegenerative diseases that arise from prolonged impaired protein folding, such as Alzheimer’s, Parkinson’s and Huntington’s disease, the UPR is not always sufficient to rescue the cell and apoptosis will be induced. Prolonged ER stress is known to activate several kinases, including glycogen synthase kinase-3β [116] and inositol-requiring kinase 1 (IRE1) which activates apoptosis signal-regulating kinase 1 (ASK1) that, in turn, stimulates c-Jun N-terminal kinase (JNK) [113, 117], ultimately leading to caspase activation and cell death. Post-mitotic neurons are especially susceptible to ER stress, as they are not protected from the accumulation of misfolded proteins through the dilution of the ER following cell division [118]. This may also account for the higher percentage of cell death observed in ΔES-transfected primary hippocampal neurons (i.e., 60 %) compared to immortalized AP-1 fibroblastic cells (i.e., ~30 %), at least under our experimental conditions.

## Conclusions

To conclude, our results provide new insight into the molecular mechanisms by which disruption of NHE6 activity impairs recycling endosomal trafficking and promotes neurodegeneration in the context of CS. These analyses provide a framework for future investigations of other NHE6 mutations and potential avenues for therapeutic interventions aimed at modulating the trafficking of NHE6-dependent recycling endosomal cargo, such as TrkB and AMPAR, thereby mitigating cell dysfunction and damage in CS. These findings may also be relevant to our understanding of other neurodevelopmental or neurodegenerative disorders such as autism [119–123], fragile X syndrome [124, 125] and Alzheimer’s disease [126] where aberrations in recycling endosomal-associated cargo and signaling events have been implicated as contributing factors.

## Additional file

Additional file 1: Supplementary data. (PDF 915 kb)

## Abbreviations

7-AAD: 7-amino actinomycin D
AMPAR: α-amino-3-hydroxy-5-methyl-4-isoxazolepropionic acid receptor
AP-1: Chinese hamster ovary (CHO) cells deficient in the Na^+^/H^+^ antiporter 1 isoform
AP2: Adaptor protein 2
Arf6: ADP-ribosylation factor 6
ARNO: ADP-ribosylation factor nucleotide site opener
AS: Angelman syndrome
ASK1: Apoptosis signal-regulating kinase 1
BDNF: Brain-derived neurotrophic factor
cCASP3: Cleaved caspase 3 (Asp175)
ChFP: Monomeric Cherry fluorescent protein
CS: Christianson syndrome
DMSO: Dimethylsulfoxide
EEA1: Early endosome antigen 1
EGF: Epidermal growth factor
EGFR: Epidermal growth factor receptor
EndoH: Endoglycosidase H
ER: Endoplasmic reticulum
ERAD: Endoplasmic reticulum-associated degradation
ESCRT: Endosomal sorting complex required for transport
FITC: Fluorescein isothiocyanate
FRIA: Fluorescence ratiometric image analysis
GAPDH: Glyceraldehyde-3-phosphate dehydrogenase
GFP: Green fluorescent protein
HA: Influenza virus hemagglutinin epitope
IRE1: Inositol-requiring kinase 1
JNK: c-Jun N-terminal kinase
Lamp-2: Lysosome-associated membrane protein 2
M.I.F.: Median intensity fluorescence
NHE6/SLC9A6: Sodium/proton exchanger isoform 6
NMDAR: *N*-methyl-D-aspartate receptor
NT-4: Neurotrophin-4
p75^NTR^: p75 neurotrophin receptor
PI: Propidium iodide
PNGaseF: Peptide-N-glycosidase F
SDS-PAGE: Sodium dodecyl sulfate polyacrylamide gel electrophoresis
siRNA: small interfering ribonucleic acids
Tf: Transferrin
TfR: Transferrin receptor
TrkB: Tropomyosin-related kinase receptor B
Ub: Ubiquitin
UBE3A/E6AP: E3 ubiquitin ligase 3A
UPR: Unfolded protein response
WT: Wild-type
XLID: X-linked intellectual disability
ΔES: Deletion of Glu287-Ser288

## References

[1] Christianson AL, Stevenson RE, van der Meyden CH, Pelser J, Theron FW, van Rensburg PL (1999). X linked severe mental retardation, craniofacial dysmorphology, epilepsy, ophthalmoplegia, and cerebellar atrophy in a large South African kindred is localised to Xq24-q27. J Med Genet.

[2] Schroer RJ, Holden KR, Tarpey PS, Matheus MG, Griesemer DA, Friez MJ (2010). Natural history of Christianson syndrome. Am J Med Genet A.

[3] Pescosolido MF, Stein DM, Schmidt M, El Moufawad AC, Sabbagh M, Rogg JM (2014). Genetic and phenotypic diversity of NHE6 mutations in Christianson syndrome. Ann Neurol.

[4] Angelman H (1961). Syndrome of coloboma with multiple congenital abnormalities in infancy. Br Med J.

[5] Williams CA, Angelman H, Clayton-Smith J, Driscoll DJ, Hendrickson JE, Knoll JH (1995). Angelman syndrome: consensus for diagnostic criteria. Angelman syndrome foundation. Am J Med Genet.

[6] Sutcliffe JS, Jiang YH, Galijaard RJ, Matsuura T, Fang P, Kubota T (1997). The E6-Ap ubiquitin-protein ligase (UBE3A) gene is localized within a narrowed Angelman syndrome critical region. Genome Res.

[7] Kishino T, Lalande M, Wagstaff J (1997). UBE3A/E6-AP mutations cause Angelman syndrome. Nat Genet.

[8] Gilfillan GD, Selmer KK, Roxrud I, Smith R, Kyllerman M, Eiklid K (2008). SLC9A6 mutations cause X-linked mental retardation, microcephaly, epilepsy, and ataxia, a phenotype mimicking Angelman syndrome. Am J Hum Genet.

[9] Garbern JY, Neumann M, Trojanowski JQ, Lee VM, Feldman G, Norris JW (2010). A mutation affecting the sodium/proton exchanger, SLC9A6, causes mental retardation with tau deposition. Brain.

[10] Tarpey PS, Smith R, Pleasance E, Whibley A, Edkins S, Hardy C (2009). A systematic, large-scale resequencing screen of X-chromosome coding exons in mental retardation. Nat Genet.

[11] Schuurs-Hoeijmakers JH, Vulto-van Silfhout AT, Vissers LE, van de Vondervoort II, van Bon BW, de Ligt J (2013). Identification of pathogenic gene variants in small families with intellectually disabled siblings by exome sequencing. J Med Genet.

[12] Tzschach A, Grasshoff U, Beck-Woedl S, Dufke C, Bauer C, Kehrer M, et al. Next-generation sequencing in X-linked intellectual disability. Eur J Hum Genet. 2015. 10.1038/ejhg.2015.5 PMC4613482 25649377

[13] Inlow JK, Restifo LL (2004). Molecular and comparative genetics of mental retardation. Genetics.

[14] Vaillend C, Poirier R, Laroche S (2008). Genes, plasticity and mental retardation. Behav Brain Res.

[15] Gécz J, Shoubridge C, Corbett M (2009). The genetic landscape of intellectual disability arising from chromosome X. Trends Genet.

[16] Ropers HH (2010). Genetics of early onset cognitive impairment. Annu Rev Genomics Hum Genet.

[17] Lubs HA, Stevenson RE, Schwartz CE (2012). Fragile X and X-linked intellectual disability: four decades of discovery. Am J Hum Genet.

[18] Schwede M, Garbett K, Mirnics K, Geschwind DH, Morrow EM (2013). Genes for endosomal NHE6 and NHE9 are misregulated in autism brains. Mol Psychiatry.

[19] Brett CL, Donowitz M, Rao R (2005). Evolutionary origins of eukaryotic sodium/proton exchangers. Am J Physiol Cell Physiol.

[20] Orlowski J, Grinstein S (2011). Na^+^/H^+^ exchangers. Compr Physiol.

[21] Miyazaki E, Sakaguchi M, Wakabayashi S, Shigekawa M, Mihara K (2001). NHE6 protein possesses a signal peptide destined for endoplasmic reticulum membrane and localizes in secretory organelles of the cell. J Biol Chem.

[22] Brett CL, Wei Y, Donowitz M, Rao R (2002). Human Na^+^/H^+^ exchanger isoform 6 is found in recycling endosomes of cells, not in mitochondria. Am J Physiol Cell Physiol.

[23] Nakamura N, Tanaka S, Teko Y, Mitsui K, Kanazawa H (2005). Four Na^+^/H^+^ exchanger isoforms are distributed to Golgi and post-Golgi compartments and are involved in organelle pH regulation. J Biol Chem.

[24] Ohgaki R, Matsushita M, Kanazawa H, Ogihara S, Hoekstra D, Van IJzendoorn SC (2010). The Na^+^/H^+^ exchanger NHE6 in the endosomal recycling system is involved in the development of apical bile canalicular surface domains in HepG2 cells. Mol Biol Cell.

[25] Xinhan L, Matsushita M, Numaza M, Taguchi A, Mitsui K, Kanazawa H (2011). Na^+^/H^+^ exchanger isoform 6 (NHE6/SLC9A6) is involved in clathrin-dependent endocytosis of transferrin. Am J Physiol Cell Physiol.

[26] Stromme P, Dobrenis K, Sillitoe RV, Gulinello M, Ali NF, Davidson C (2011). X-linked Angelman-like syndrome caused by Slc9a6 knockout in mice exhibits evidence of endosomal-lysosomal dysfunction. Brain.

[27] Deane EC, Ilie AE, Sizdahkhani S, Das Gupta M, Orlowski J, McKinney RA (2013). Enhanced recruitment of endosomal Na^+^/H^+^ Exchanger NHE6 into dendritic spines of hippocampal pyramidal neurons during NMDA receptor-dependent long-term potentiation. J Neurosci.

[28] Ouyang Q, Lizarraga SB, Schmidt M, Yang U, Gong J, Ellisor D (2013). Christianson syndrome protein NHE6 modulates TrkB endosomal signaling required for neuronal circuit development. Neuron.

[29] Lein ES, Hawrylycz MJ, Ao N, Ayres M, Bensinger A, Bernard A (2007). Genome-wide atlas of gene expression in the adult mouse brain. Nature.

[30] Lu W, Man H, Ju W, Trimble WS, MacDonald JF, Wang YT (2001). Activation of synaptic NMDA receptors induces membrane insertion of new AMPA receptors and LTP in cultured hippocampal neurons. Neuron.

[31] Huang SH, Wang J, Sui WH, Chen B, Zhang XY, Yan J (2013). BDNF-dependent recycling facilitates TrkB translocation to postsynaptic density during LTP via a Rab11-dependent pathway. J Neurosci.

[32] Hu H, Wrogemann K, Kalscheuer V, Tzschach A, Richard H, Haas SA (2009). Mutation screening in 86 known X-linked mental retardation genes by droplet-based multiplex PCR and massive parallel sequencing. Hugo J.

[33] Madrigal I, Fernandez-Burriel M, Rodriguez-Revenga L, Cabrera JC, Marti M, Mur A, Mila M (2010). Xq26.2-q26.3 microduplication in two brothers with intellectual disabilities: clinical and molecular characterization. J Hum Genet.

[34] Takahashi Y, Hosoki K, Matsushita M, Funatsuka M, Saito K, Kanazawa H (2011). A loss-of-function mutation in the SLC9A6 gene causes X-linked mental retardation resembling Angelman syndrome. Am J Med Genet B Neuropsychiatr Genet.

[35] Tzschach A, Ullmann R, Ahmed A, Martin T, Weber G, Decker-Schwering O (2011). Christianson syndrome in a patient with an interstitial Xq26.3 deletion. Am J Med Genet A.

[36] Piton A, Gauthier J, Hamdan FF, Lafreniere RG, Yang Y, Henrion E (2011). Systematic resequencing of X-chromosome synaptic genes in autism spectrum disorder and schizophrenia. Mol Psychiatry.

[37] Riess A, Rossier E, Kruger R, Dufke A, Beck-Woedl S, Horber V (2013). Novel SLC9A6 mutations in two families with Christianson syndrome. Clin Genet.

[38] Bosemani T, Zanni G, Hartman AL, Cohen R, Huisman TA, Bertini E, Poretti A (2014). Christianson syndrome: spectrum of neuroimaging findings. Neuropediatrics.

[39] Mignot C, Heron D, Bursztyn J, Momtchilova M, Mayer M, Whalen S (2013). Novel mutation in SLC9A6 gene in a patient with Christianson syndrome and retinitis pigmentosum. Brain Dev.

[40] Zanni G, Barresi S, Cohen R, Specchio N, Basel-Vanagaite L, Valente EM (2014). A novel mutation in the endosomal Na^+^/H^+^ exchanger NHE6 (SLC9A6) causes Christianson syndrome with electrical status epilepticus during slow-wave sleep (ESES). Epilepsy Res.

[41] Redin C, Gerard B, Lauer J, Herenger Y, Muller J, Quartier A (2014). Efficient strategy for the molecular diagnosis of intellectual disability using targeted high-throughput sequencing. J Med Genet.

[42] Ilie A, Weinstein E, Boucher A, McKinney RA, Orlowski J (2014). Impaired posttranslational processing and trafficking of an endosomal Na^+^/H^+^ exchanger NHE6 mutant (D^370^WST^372^) associated with X-linked intellectual disability and autism. Neurochem Int.

[43] Roxrud I, Raiborg C, Gilfillan GD, Stromme P, Stenmark H (2009). Dual degradation mechanisms ensure disposal of NHE6 mutant protein associated with neurological disease. Exp Cell Res.

[44] Rotin D, Grinstein S (1989). Impaired cell volume regulation in Na^+^-H^+^ exchange-deficient mutants. Am J Physiol.

[45] Barriere H, Lukacs GL (2008). Analysis of endocytic trafficking by single-cell fluorescence ratio imaging. Curr Protoc Cell Biol.

[46] Jiang M, Chen G (2006). High Ca^2+^-phosphate transfection efficiency in low-density neuronal cultures. Nat Protoc.

[47] Porter K, Prescott D, Frye J (1973). Changes in surface morphology of Chinese hamster ovary cells during the cell cycle. J Cell Biol.

[48] Fafournoux P, Noël J, Pouysségur J (1994). Evidence that Na^+^/H^+^ exchanger isoforms NHE1 and NHE3 exist as stable dimers in membranes with a high degree of specificity for homodimers. J Biol Chem.

[49] Hisamitsu T, Pang T, Shigekawa M, Wakabayashi S (2004). Dimeric interaction between the cytoplasmic domains of the Na^+^/H^+^ exchanger NHE1 revealed by symmetrical intermolecular cross-linking and selective co-immunoprecipitation. Biochemistry.

[50] Hisamitsu T, Ammar YB, Nakamura TY, Wakabayashi S (2006). Dimerization Is Crucial for the Function of the Na^+^/H^+^ Exchanger NHE1. Biochemistry.

[51] Meusser B, Hirsch C, Jarosch E, Sommer T (2005). ERAD: the long road to destruction. Nat Cell Biol.

[52] Raasi S, Wolf DH (2007). Ubiquitin receptors and ERAD: a network of pathways to the proteasome. Semin Cell Dev Biol.

[53] Raiborg C, Stenmark H (2009). The ESCRT machinery in endosomal sorting of ubiquitylated membrane proteins. Nature.

[54] Apaja PM, Xu H, Lukacs GL (2010). Quality control for unfolded proteins at the plasma membrane. J Cell Biol.

[55] Goder V (2012). Roles of ubiquitin in endoplasmic reticulum-associated protein degradation (ERAD). Curr Protein Pept Sci.

[56] Barriere H, Nemes C, Du K, Lukacs GL (2007). Plasticity of polyubiquitin recognition as lysosomal targeting signals by the endosomal sorting machinery. Mol Biol Cell.

[57] Piper RC, Dikic I, Lukacs GL. Ubiquitin-dependent sorting in endocytosis. Cold Spring Harb Perspect Biol. 2014;6. 10.1101/cshperspect.a016808 PMC3941215 24384571

[58] Le Bivic A, Real FX, Rodriguez-Boulan E (1989). Vectorial targeting of apical and basolateral plasma membrane proteins in a human adenocarcinoma epithelial cell line. Proc Natl Acad Sci U S A.

[59] Dayel MJ, Hom EF, Verkman AS (1999). Diffusion of green fluorescent protein in the aqueous-phase lumen of endoplasmic reticulum. Biophys J.

[60] Wilson JM, de Hoop M, Zorzi N, Toh BH, Dotti CG, Parton RG (2000). EEA1, a tethering protein of the early sorting endosome, shows a polarized distribution in hippocampal neurons, epithelial cells, and fibroblasts. Mol Biol Cell.

[61] Vanlandingham PA, Ceresa BP (2009). Rab7 regulates late endocytic trafficking downstream of multivesicular body biogenesis and cargo sequestration. J Biol Chem.

[62] Glozman R, Okiyoneda T, Mulvihill CM, Rini JM, Barriere H, Lukacs GL (2009). N-glycans are direct determinants of CFTR folding and stability in secretory and endocytic membrane traffic. J Cell Biol.

[63] Sigismund S, Woelk T, Puri C, Maspero E, Tacchetti C, Transidico P (2005). Clathrin-independent endocytosis of ubiquitinated cargos. Proc Natl Acad Sci U S A.

[64] Sigismund S, Argenzio E, Tosoni D, Cavallaro E, Polo S, Di Fiore PP (2008). Clathrin-mediated internalization is essential for sustained EGFR signaling but dispensable for degradation. Dev Cell.

[65] Kazazic M, Roepstorff K, Johannessen LE, Pedersen NM, van Deurs B, Stang E, Madshus IH (2006). EGF-induced activation of the EGF receptor does not trigger mobilization of caveolae. Traffic.

[66] Johnson LS, Dunn KW, Pytowski B, McGraw TE (1993). Endosome acidification and receptor trafficking: bafilomycin A1 slows receptor externalization by a mechanism involving the receptor’s internalization motif. Mol Biol Cell.

[67] Weisz OA (2003). Acidification and protein traffic. Int Rev Cytol.

[68] Casey JR, Grinstein S, Orlowski J (2010). Sensors and regulators of intracellular pH. Nat Rev Mol Cell Biol.

[69] Barriere H, Apaja P, Okiyoneda T, Lukacs GL (2011). Endocytic sorting of CFTR variants monitored by single-cell fluorescence ratiometric image analysis (FRIA) in living cells. Methods Mol Biol.

[70] Hacker G (2000). The morphology of apoptosis. Cell Tissue Res.

[71] Maeno E, Ishizaki Y, Kanaseki T, Hazama A, Okada Y (2000). Normotonic cell shrinkage because of disordered volume regulation is an early prerequisite to apoptosis. Proc Natl Acad Sci U S A.

[72] Bortner CD, Cidlowski JA (2004). The role of apoptotic volume decrease and ionic homeostasis in the activation and repression of apoptosis. Pflugers Arch.

[73] Andree HA, Reutelingsperger CP, Hauptmann R, Hemker HC, Hermens WT, Willems GM (1990). Binding of vascular anticoagulant alpha (VAC alpha) to planar phospholipid bilayers. J Biol Chem.

[74] Vandenabeele P, Galluzzi L, Vanden Berghe T, Kroemer G (2010). Molecular mechanisms of necroptosis: an ordered cellular explosion. Nat Rev Mol Cell Biol.

[75] Vermes I, Haanen C, Steffens-Nakken H, Reutelingsperger C (1995). A novel assay for apoptosis. Flow cytometric detection of phosphatidylserine expression on early apoptotic cells using fluorescein labelled Annexin V. J Immunol Methods.

[76] Galluzzi L, Aaronson SA, Abrams J, Alnemri ES, Andrews DW, Baehrecke EH (2009). Guidelines for the use and interpretation of assays for monitoring cell death in higher eukaryotes. Cell Death Differ.

[77] Galluzzi L, Bravo-San Pedro JM, Vitale I, Aaronson SA, Abrams JM, Adam D (2015). Essential versus accessory aspects of cell death: recommendations of the NCCD 2015. Cell Death Differ.

[78] Sharma M, Pampinella F, Nemes C, Benharouga M, So J, Du K (2004). Misfolding diverts CFTR from recycling to degradation: quality control at early endosomes. J Cell Biol.

[79] Okiyoneda T, Barriere H, Bagdany M, Rabeh WM, Du K, Hohfeld J (2010). Peripheral protein quality control removes unfolded CFTR from the plasma membrane. Science.

[80] Apaja PM, Foo B, Okiyoneda T, Valinsky WC, Barriere H, Atanasiu R (2013). Ubiquitination-dependent quality control of hERG K^+^ channel with acquired and inherited conformational defect at the plasma membrane. Mol Biol Cell.

[81] Motley A, Bright NA, Seaman MN, Robinson MS (2003). Clathrin-mediated endocytosis in AP-2-depleted cells. J Cell Biol.

[82] Kazazic M, Bertelsen V, Pedersen KW, Vuong TT, Grandal MV, Rodland MS (2009). Epsin 1 is involved in recruitment of ubiquitinated EGF receptors into clathrin-coated pits. Traffic.

[83] Jiang X, Huang F, Marusyk A, Sorkin A (2003). Grb2 regulates internalization of EGF receptors through clathrin-coated pits. Mol Biol Cell.

[84] Johannessen LE, Pedersen NM, Pedersen KW, Madshus IH, Stang E (2006). Activation of the epidermal growth factor (EGF) receptor induces formation of EGF receptor- and Grb2-containing clathrin-coated pits. Mol Cell Biol.

[85] Yamashiro DJ, Tycko B, Fluss SR, Maxfield FR (1984). Segregation of transferrin to a mildly acidic (pH 6.5) para-Golgi compartment in the recycling pathway. Cell.

[86] Mellman I, Fuchs R, Helenius A (1986). Acidification of the endocytic and exocytic pathways. Annu Rev Biochem.

[87] Maxfield FR, McGraw TE (2004). Endocytic recycling. Nat Rev Mol Cell Biol.

[88] Ding J, Rainey JK, Xu C, Sykes BD, Fliegel L (2006). Structural and functional characterization of transmembrane segment VII of the Na^+^/H^+^ exchanger isoform 1. J Biol Chem.

[89] van Weert AW, Dunn KW, Gueze HJ, Maxfield FR, Stoorvogel W (1995). Transport from late endosomes to lysosomes, but not sorting of integral membrane proteins in endosomes, depends on the vacuolar proton pump. J Cell Biol.

[90] Hurtado-Lorenzo A, Skinner M, El AJ, Futai M, Sun-Wada GH, Bourgoin S (2006). V-ATPase interacts with ARNO and Arf6 in early endosomes and regulates the protein degradative pathway. Nat Cell Biol.

[91] Merkulova M, Bakulina A, Thaker YR, Gruber G, Marshansky V (1797). Specific motifs of the V-ATPase a2-subunit isoform interact with catalytic and regulatory domains of ARNO. Biochim Biophys Acta.

[92] Souza-Schorey C, van Donselaar E, Hsu VW, Yang C, Stahl PD, Peters PJ (1998). ARF6 targets recycling vesicles to the plasma membrane: insights from an ultrastructural investigation. J Cell Biol.

[93] Radhakrishna H, Al-Awar O, Khachikian Z, Donaldson JG (1999). ARF6 requirement for Rac ruffling suggests a role for membrane trafficking in cortical actin rearrangements. J Cell Sci.

[94] Prigent M, Dubois T, Raposo G, Derrien V, Tenza D, Rosse C (2003). ARF6 controls post-endocytic recycling through its downstream exocyst complex effector. J Cell Biol.

[95] Taylor RC, Cullen SP, Martin SJ (2008). Apoptosis: controlled demolition at the cellular level. Nat Rev Mol Cell Biol.

[96] Galluzzi L, Bravo-San Pedro JM, Kroemer G (2014). Organelle-specific initiation of cell death. Nat Cell Biol.

[97] Bredesen DE, Rao RV, Mehlen P (2006). Cell death in the nervous system. Nature.

[98] Park M, Salgado JM, Ostroff L, Helton TD, Robinson CG, Harris KM, Ehlers MD (2006). Plasticity-induced growth of dendritic spines by exocytic trafficking from recycling endosomes. Neuron.

[99] Petrini EM, Lu J, Cognet L, Lounis B, Ehlers MD, Choquet D (2009). Endocytic trafficking and recycling maintain a pool of mobile surface AMPA receptors required for synaptic potentiation. Neuron.

[100] Walkley SU, Vanier MT (1793). Secondary lipid accumulation in lysosomal disease. Biochim Biophys Acta.

[101] Cao C, Rioult-Pedotti MS, Migani P, Yu CJ, Tiwari R, Parang K (2013). Impairment of TrkB-PSD-95 Signaling in Angelman Syndrome. PLoS Biol.

[102] Ip NY, Li Y, Yancopoulos GD, Lindsay RM (1993). Cultured hippocampal neurons show responses to BDNF, NT-3, and NT-4, but not NGF. J Neurosci.

[103] Leal G, Afonso PM, Salazar IL, Duarte CB (1621). Regulation of hippocampal synaptic plasticity by BDNF. Brain Res.

[104] Huang EJ, Reichardt LF (2001). Neurotrophins: roles in neuronal development and function. Annu Rev Neurosci.

[105] Coulson EJ, Reid K, Bartlett PF (1999). Signaling of neuronal cell death by the p75NTR neurotrophin receptor. Mol Neurobiol.

[106] Zagrebelsky M, Holz A, Dechant G, Barde YA, Bonhoeffer T, Korte M (2005). The p75 neurotrophin receptor negatively modulates dendrite complexity and spine density in hippocampal neurons. J Neurosci.

[107] Friedman WJ (2000). Neurotrophins induce death of hippocampal neurons via the p75 receptor. J Neurosci.

[108] Troy CM, Friedman JE, Friedman WJ (2002). Mechanisms of p75-mediated death of hippocampal neurons. Role of caspases. J Biol Chem.

[109] Schenck A, Goto-Silva L, Collinet C, Rhinn M, Giner A, Habermann B (2008). The endosomal protein Appl1 mediates Akt substrate specificity and cell survival in vertebrate development. Cell.

[110] Schutze S, Tchikov V, Schneider-Brachert W (2008). Regulation of TNFR1 and CD95 signalling by receptor compartmentalization. Nat Rev Mol Cell Biol.

[111] Bredesen DE, Mehlen P, Rabizadeh S (2005). Receptors that mediate cellular dependence. Cell Death Differ.

[112] Thibert C, Fombonne J (2010). Dependence receptors: mechanisms of an announced death. Cell Cycle.

[113] Tabas I, Ron D (2011). Integrating the mechanisms of apoptosis induced by endoplasmic reticulum stress. Nat Cell Biol.

[114] Doyle KM, Kennedy D, Gorman AM, Gupta S, Healy SJ, Samali A (2011). Unfolded proteins and endoplasmic reticulum stress in neurodegenerative disorders. J Cell Mol Med.

[115] Viana RJ, Nunes AF, Rodrigues CM (2012). Endoplasmic reticulum enrollment in Alzheimer’s disease. Mol Neurobiol.

[116] Brewster JL, Linseman DA, Bouchard RJ, Loucks FA, Precht TA, Esch EA, Heidenreich KA (2006). Endoplasmic reticulum stress and trophic factor withdrawal activate distinct signaling cascades that induce glycogen synthase kinase-3 beta and a caspase-9-dependent apoptosis in cerebellar granule neurons. Mol Cell Neurosci.

[117] Urano F, Wang X, Bertolotti A, Zhang Y, Chung P, Harding HP, Ron D (2000). Coupling of stress in the ER to activation of JNK protein kinases by transmembrane protein kinase IRE1. Science.

[118] Roussel BD, Kruppa AJ, Miranda E, Crowther DC, Lomas DA, Marciniak SJ (2013). Endoplasmic reticulum dysfunction in neurological disease. Lancet Neurol.

[119] Correia CT, Coutinho AM, Sequeira AF, Sousa IG, Lourenco VL, Almeida JP (2010). Increased BDNF levels and NTRK2 gene association suggest a disruption of BDNF/TrkB signaling in autism. Genes Brain Behav.

[120] Scattoni ML, Martire A, Cartocci G, Ferrante A, Ricceri L (2013). Reduced social interaction, behavioural flexibility and BDNF signalling in the BTBR T+ tf/J strain, a mouse model of autism. Behav Brain Res.

[121] Koh JY, Lim JS, Byun HR, Yoo MH (2014). Abnormalities in the zinc-metalloprotease-BDNF axis may contribute to megalencephaly and cortical hyperconnectivity in young autism spectrum disorder patients. Mol Brain.

[122] Purcell AE, Jeon OH, Zimmerman AW, Blue ME, Pevsner J (2001). Postmortem brain abnormalities of the glutamate neurotransmitter system in autism. Neurology.

[123] Carlson GC (2012). Glutamate receptor dysfunction and drug targets across models of autism spectrum disorders. Pharmacol Biochem Behav.

[124] Lauterborn JC, Rex CS, Kramar E, Chen LY, Pandyarajan V, Lynch G, Gall CM (2007). Brain-derived neurotrophic factor rescues synaptic plasticity in a mouse model of fragile X syndrome. J Neurosci.

[125] Louhivuori V, Vicario A, Uutela M, Rantamaki T, Louhivuori LM, Castren E (2011). BDNF and TrkB in neuronal differentiation of Fmr1-knockout mouse. Neurobiol Dis.

[126] Gao L, Tian M, Zhao HY, Xu QQ, Huang YM, Si QC (2016). TrkB activation by 7, 8-dihydroxyflavone increases synapse AMPA subunits and ameliorates spatial memory deficits in a mouse model of Alzheimer’s disease. J Neurochem.

[127] Wakabayashi S, Pang T, Su X, Shigekawa M (2000). A novel topology model of the human Na^+^/H^+^ exchanger isoform 1. J Biol Chem.

[128] Nygaard EB, Lagerstedt JO, Bjerre G, Shi B, Budamagunta M, Poulsen KA (2011). Structural modeling and electron paramagnetic resonance spectroscopy of the human Na^+^/H^+^ exchanger isoform 1, NHE1. J Biol Chem.

