# Supplementary material for: A Christianson syndrome-linked deletion mutation (∆287ES288) in SLC9A6 disrupts recycling endosomal function and elicits neurodegeneration and cell death
Source: Mol Neurodegener. 2016 Sep 2;11(1):63. doi: 10.1186/s13024-016-0129-9 (PMC5010692; doi:10.1186/s13024-016-0129-9)
Supplement: Additional file 1: — Supplementary data. (PDF 915 kb) [file 13024_2016_129_MOESM1_ESM.pdf]

Additional File 1: Supplementary Data

A Christianson Syndrome-linked deletion mutation ($\Delta^{287}ES^{288}$) in SLC9A6 disrupts recycling endosomal function and elicits neurodegeneration and cell death

Alina Ilie[1], Andy Y. L. Gao[2], Jonathan Reid[1], Annie Boucher[1], Cassandra McEwan[1], Hervé Barrière[1], Gergely L. Lukacs[1], R. Anne McKinney[2] and John Orlowski[1]*

[1]Department of Physiology, and [2]Department of Pharmacology and Therapeutics, McGill University, Montreal, Canada

	M7 (280 · 290 · 300)
hNHE6	DVELYALLFGESVLNDAVAIVLSS
hNHE1	NELLHILVFGESLLNDAVTVVLYH
hNHE2	NEQLYILVFGESLLNDAVTVVLYN
hNHE3	NEVLFIIVFGESLLNDAVTVVLYN
hNHE4	NEQLYMMIFGEALLNDGITVVLYN
hNHE5	NETLFIIVFGESLLNDAVTVVLYK
hNHE7	DVDLYALLFGESVLNDAVAIVLSS
hNHE8	DPVLNMLVFGESILNDAVSIVLTN
hNHE9	DPDLYTLLFGESVLNDAVAIVLTY

Figure S1. Sequence alignment of the different human NHE isoforms in the region encompassing the E287-S288 deletion mutation (ΔES). The ES amino acids are located in the predicted seventh transmembrane helix (M7) according to the membrane topology models proposed by Wakabayashi *et al*. [1] and Nygaard *et al*. [2]

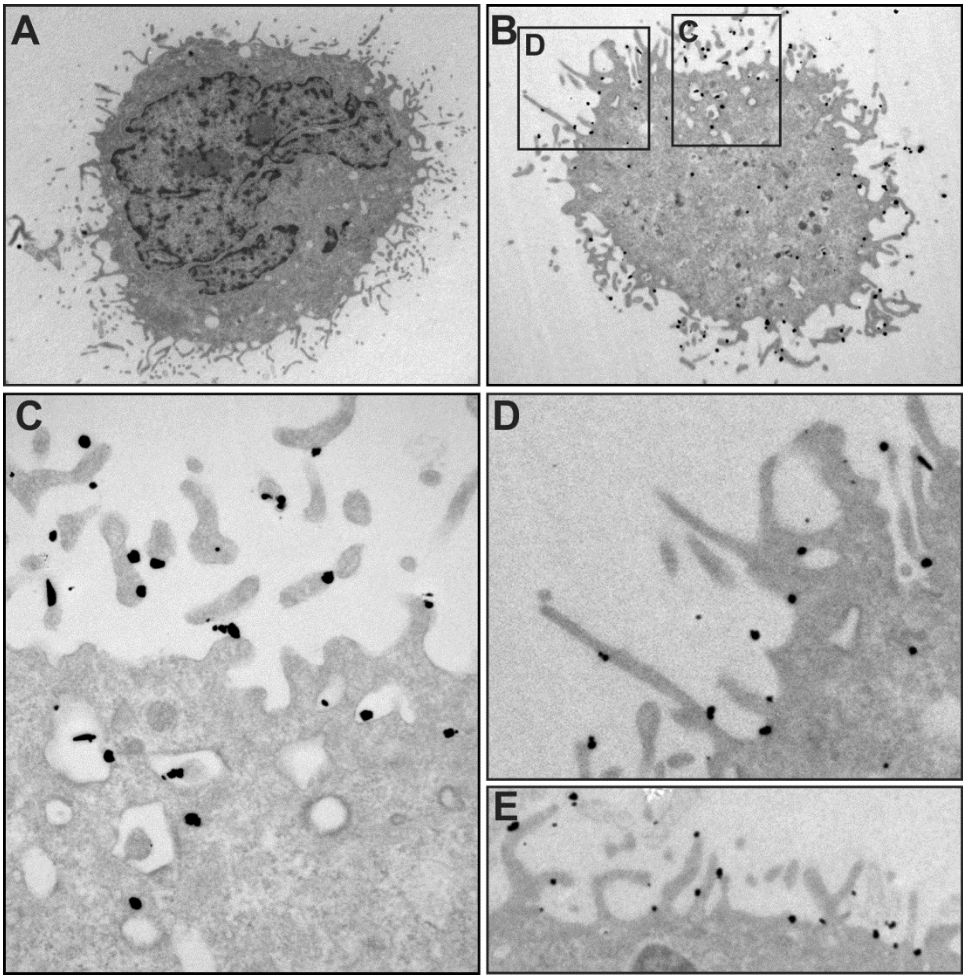

Fig. S2. Detection of NHE6 at the plasma membrane and endosomes in transfected AP-1 cells by transmission electron microscopy. AP-1 cells stably expressing human NHE6v1$_{HA}$ were incubated in the absence (negative control) (***A***) or presence (***B-D***) of the primary anti-HA antibody, followed by incubation with the secondary anti-IgG antibody conjugated to 0.8 nM gold particle. Magnification: (***A***) x4400, (***B***) x6100, (***C, D***) x24400, (***E***) x8100. Images are representative of two experiments.

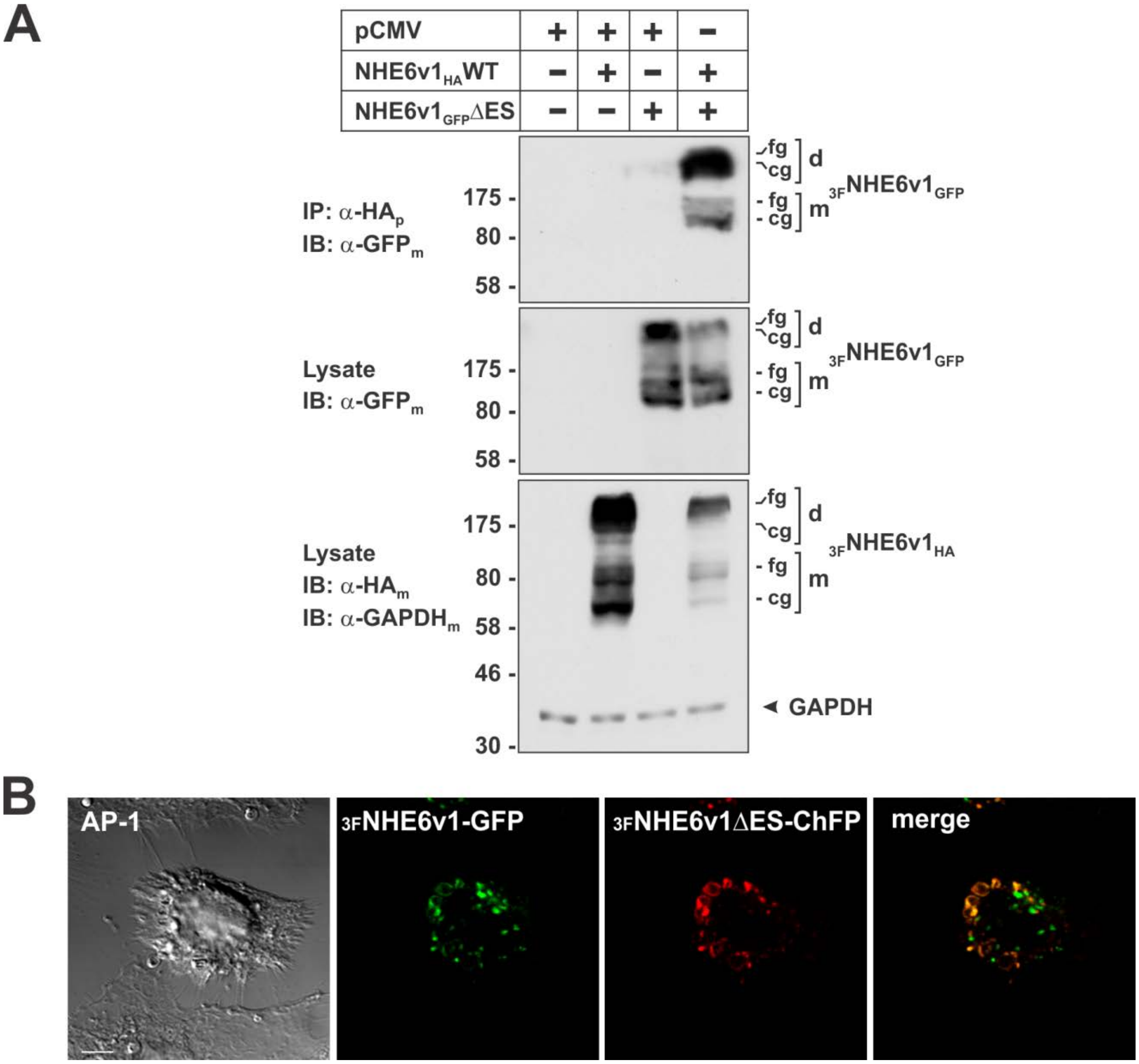

Fig. S3. NHE6v1 wild-type and ΔES can form a complex in intact cells. ***A***, HeLa cells were transiently transfected with empty vector (pCMV), NHE6v1$_{HA}$ WT or ΔES either singly or in combination and then cell lysates were prepared 24 h post-transfection. Cell lysate were incubated with a polyclonal rabbit anti-HA antibody (α-HA$_{p}$) and the immunoprecipitated proteins, as well as aliquots of initial lysates were resolved by SDS-PAGE and immunoblotting with the indicated mouse monoclonal antibodies. ***B***, AP-1 cells were transiently transfected with $_{3F}$NHE6v1$_{GFP}$-WT and $_{3F}$NHE6v1$_{ChFP}$-ΔES and then imaged by confocal microscopy. The WT and ΔES constructs show partial colocalization. *Scale bars* represent 5 µm. Data are representative of three experiments.

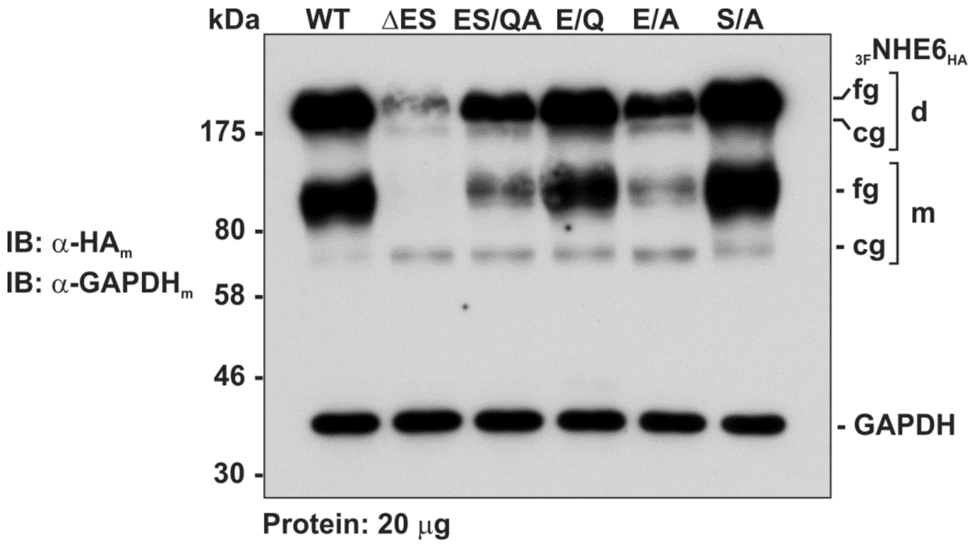

Fig. S4. Expression of wild-type and mutant NHE6v1 constructs in transfected AP-1 cells. AP-1 cells were transiently transfected with WT and various mutant forms (ΔES, ES/QA, E/Q, E/A, S/A) of $NHE6_{HA}$. Total cell lysates were prepared 48 h post-transfection and analyzed by Western blotting with a mouse monoclonal anti-HA antibody (α-HA_m). The same membrane was immunoblotted with a mouse monoclonal anti-GAPDH antibody (α-$GAPDH_m$) as a control for protein loading. Results are representative of three experiments.

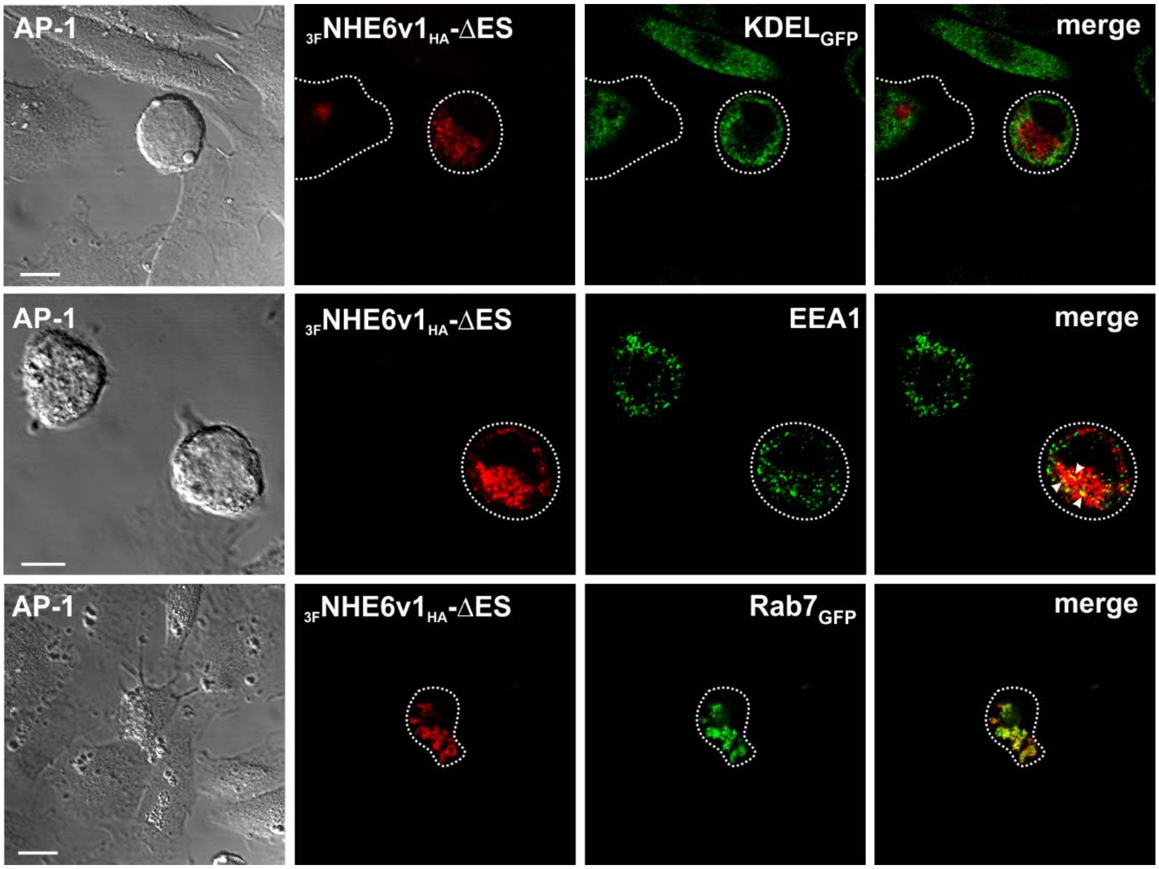

Fig. S5. Subcellular localization of NHE6v1 ΔES in transfected AP-1 cells. AP-1 cells were transiently cotransfected (24 h) with $_{3F}$NHE6v1$_{HA}$ ΔES and markers for the endoplasmic reticulum (KDEL$_{GFP}$) and late endosomes/multivesicular bodies (Rab7$_{GFP}$) or dual immunolabelled for endogenous early endosomal antigen 1 (EEA1). Footprints of the transfected cells are indicated as white dotted outlines and were derived from the transmitted light images (*far left panels*). Arrowheads in the merged image for EEA1 indicate areas of partial overlap with the ΔES mutant. Results are representative of three experiments.

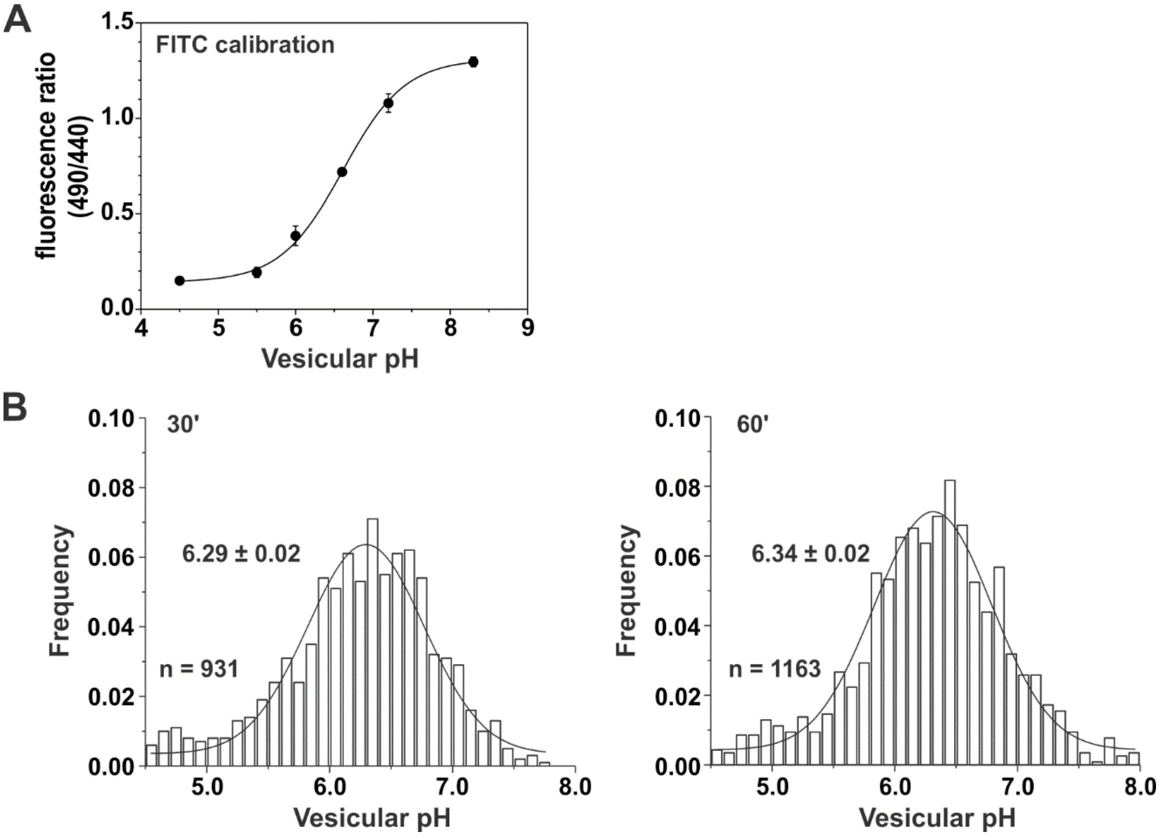

Fig. S6. Measurement of recycling endosomal pH in AP-1 cells. FITC-conjugated transferrin (Tf-FITC) (10 µg/ml) was incubated with live untransfected AP-1 cells for 1 h on ice. The temperature was then raised to 37 °C for 30 or 60 min and endosomal pH was measured by fluorescence ratio imaging (FRIA) as described in "Methods". ***A***, *In situ* calibration of Tf-FITC fluorescence as a function of vesicular pH was performed by clamping the vesicular pH between 4.5 and 8.3 as described in "Methods". ***B***, Measurement of vesicular pH as a function of time (30 and 60 min) following internalization of Tf-FITC. The pH values represent the mean ± S.E.M. of the total number of vesicles (n) from 3 to 4 cells per field of view (~300 vesicles/cell) analyzed from a representative experiment.

REFERENCES

1. Wakabayashi S, Pang T, Su X, Shigekawa M. A novel topology model of the human Na^+/H^+ exchanger isoform 1**.** J Biol Chem. 2000;275**:**7942-7949.
2. Nygaard EB, Lagerstedt JO, Bjerre G, Shi B, Budamagunta M, Poulsen KA et al. Structural modeling and electron paramagnetic resonance spectroscopy of the human Na^+/H^+ exchanger isoform 1, NHE1**.** J Biol Chem. 2011;286**:**634-648.
